# Ethnobotany of the Aegadian Islands: safeguarding biocultural refugia in the Mediterranean

**DOI:** 10.1186/s13002-021-00470-z

**Published:** 2021-07-28

**Authors:** Alfonso La Rosa, Laura Cornara, Alessandro Saitta, Akram M. Salam, Santo Grammatico, Marco Caputo, Tommaso La Mantia, Cassandra L. Quave

**Affiliations:** 1 Cooperativa Silene, Via D’Ondes Reggio 8/A, 90100 Palermo, Italy; 2 Department of Earth, Environment and Life Sciences, University of Genova, Corso Europa 26, 16132 Genova, Italy; 3 Department of Agricultural, Food and Forest Sciences, University of Palermo, Viale delle Scienze, Bld. 4, 90128 Palermo, Italy; 4 Molecular and Systems Pharmacology Program, Emory University, 615 Michael St., Whitehead 115, Atlanta, GA 30322 USA; 5 Legambiente Liguria Onlus, Via Caffa 3/5, 16129 Genova, Italy; 6 Center for the Study of Human Health, Emory University, 1557 Dickey Dr, Anthropology 306, Atlanta, GA 30322 USA; 7 Department of Dermatology, Emory University School of Medicine, 615 Michael St., Whitehead 105L, Atlanta, GA 30322 USA

**Keywords:** The Mediterranean, Medicinal plants, *Glaucium flavum*, *Agave sisalana*, *Pleurotus eryngii*, *Artemisia arborescens*, *Ruta chalepensis*

## Abstract

**Background:**

The Aegadian Islands are located west of Trapani, Sicily. Once the site of bountiful tuna fisheries and fruit orchards (plums, peaches, apricots), grapevines, prickly pears, and grains, the local economy is now based on tourism, and many traditional agricultural and maritime practices have been abandoned. In this study, we aimed to evaluate the state of traditional ecological knowledge (TEK) concerning the use of wild and cultivated plants and fungi for human health, food, maritime, and agricultural purposes on the islands of Levanzo, Favignana, and Marettimo and compare present-day practices with those documented in the past.

**Methods:**

In-depth semi-structured interviews were conducted in Italian with 48 participants with prior informed consent from May 2016 to July 2017 and October 2018. Herbarium voucher specimens of wild species were collected for herbarium deposit. A rigorous literature review of scientific and other local reports on TEK of wild flora and their application in food, health, and household applications was undertaken for the purpose of comparing findings from this field study with prior reports.

**Results:**

A total of 122 plant and five fungal taxa representing 54 families were cited for 355 uses. Among the most pervasive species in the landscape, *Agave americana* and *A. sisalana* had diverse applications in the past, which ranged from cordage for agricultural and maritime applications to tools for sewing, eating land snails, and constructing furniture. Fields of *Ferula communis* also dominate the landscape, and the dry stems were used extensively in furniture making; this species also serves as an environmental indicator for the location of the most preferred edible mushrooms, *Pleurotus eryngii* var. *ferulae*. Other important flora included topical medicinal applications of *Glaucium flavum* for hematomas and *Artemisia arborescens* for ritual bathing of newborns.

**Conclusion:**

While many plant-based traditions have disappeared from daily practice, especially those related to traditional fishing and health practices, they remain in the memories of the eldest subset of the population. Documenting this knowledge before it disappears from oral history is a key factor in reducing loss of TEK and biocultural diversity, safeguarding the role of the Aegadian Islands as biocultural refugia.

**Supplementary Information:**

The online version contains supplementary material available at 10.1186/s13002-021-00470-z.

## Background

The Mediterranean region encompasses three continents (Europe, Asia, and Africa) and has a range of topographic features from mountains, scrublands, coastal wetlands, forests, woodlands, savannas, grasslands islands, and more. The Mediterranean is recognized as one of the major biodiversity hotspots in the world, with a floral diversity of 25,000 species, and 60% of which is unique to the region. At the same time, local flora are under serious threat, and many species may be lost even in this century. According to the IUCN Red List of Threatened Species report, habitat loss and coastal infrastructural development are major contributors to the decline of biodiversity in the region [1]. In addition to biodiversity, there is an incredibly rich amount of biocultural heritage due to the myriad languages and cultural exchanges that have taken place here over the centuries, which have fostered significant diversification of cultural practices regarding the uses of wild species as sources of food, medicine, tools and more. In order to capture a high level of biocultural diversity, as well as sample from diverse plant populations, Quave and colleagues have undertaken a series of ethnobotanical field studies throughout the region, with a special emphasis on islands and mountains as biocultural refugia of traditional ecological knowledge (TEK) concerning local flora (for example, see [2–9]). Biocultural refugia have been defined as “places that not only shelter species, but also carry knowledge and experiences about the practical management of biodiversity and ecosystem services” [10].

### History and agronomic transformations of the landscape

**Fig. 1:**
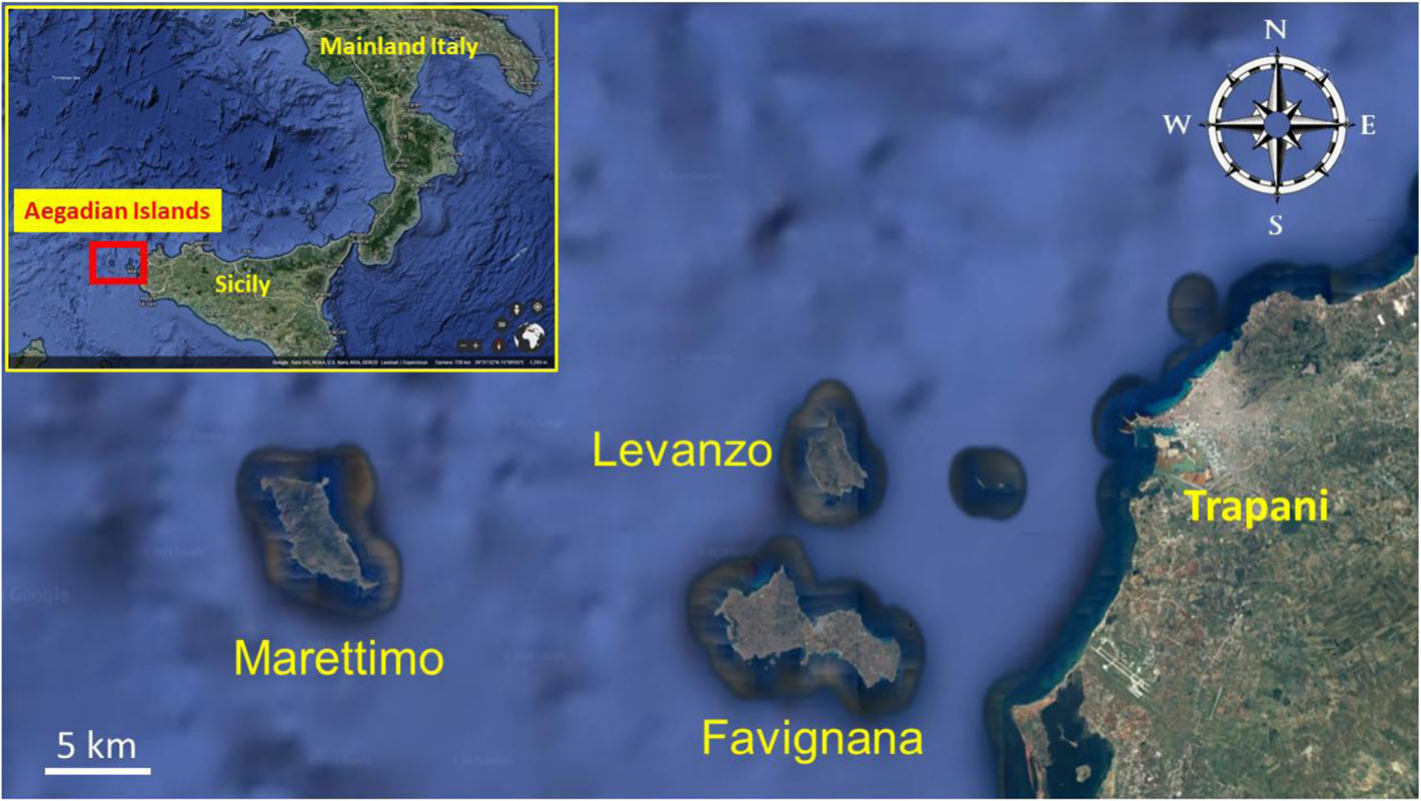
Map of the study area. Image adapted from Google Earth (https://earth.google.com/)

The Aegadian Islands (Fig. 1) have undergone ancient agronomic transformations since the time of the Phoenicians and Romans. However, complex historical events related to piracy prevented the spread of agriculture across the three islands until the beginning of the 18th century [11, 12]. This can be traced back to 1637, when the noble Pallavicino family bought the islands [13].

Local agricultural systems on these islands developed with some unique characteristics but the process of genetic erosion, closely associated with land abandonment, has unfortunately impacted the Aegadian islands, where the limited agricultural terrain makes this process faster. While cereal grains and local horticultural varieties proved to be particularly vulnerable to economic changes in the islands, fruit diversity persists as a living witness of agroecological diversity and in this sense, every small circum-Sicilian island has its own history [14, 15].

**Fig. 2:**
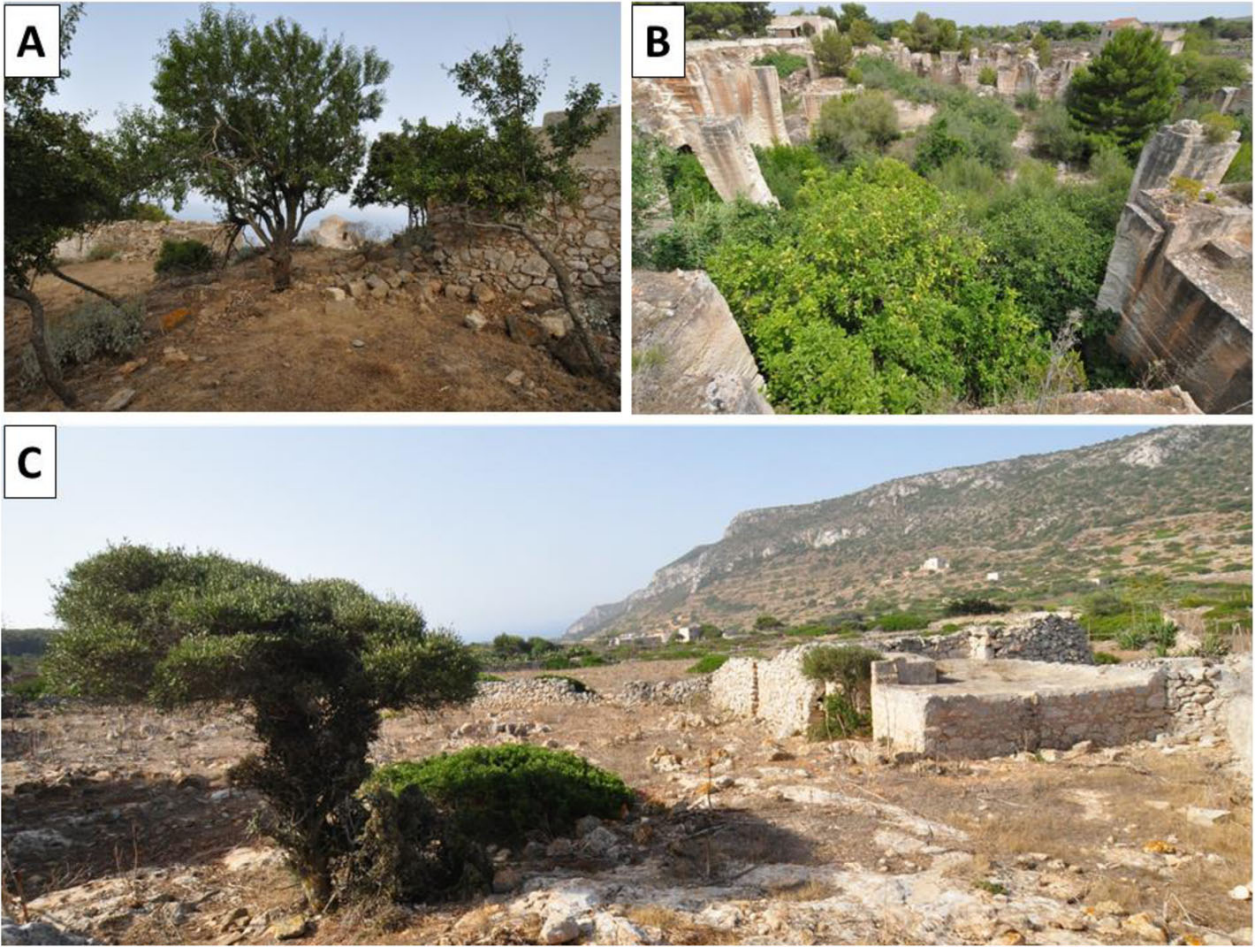
Characteristic landscapes of the Aegadian Islands. **A** In Marettimo, only small strips of orchards remain, including those of the ancient Roman houses where plum, mulberry, and almond trees can be found near the archeological remains of the site. These remnants represent a unique testimony to the history and biocultural refugia of this landscape. **B** The unique agriculture of Favignana, even if largely abandoned today, has extraordinary landscape characteristics because the orchards were cultivated inside limestone tuff quarries, which offer excellent shelter from the wind. The plants remaining in the quarries today are a precious genetic heritage to be preserved. **C** In Levanzo, agriculture has been totally abandoned. Yet, in the countryside, artifacts and plants (e.g., fig and olive trees) bear witness to the history of widespread cultivation of the landscape

Of the three main Aegadian islands, Marettimo—formerly known as Hiera, part of the Greek name for “sacred island”—is the most distant from the mainland and is considered to be the most "wild," where the few traces of agriculture still evident are linked to the historic Roman houses and around the close borders of the town (Fig. 2A). Giuffrida [13] reports at the end of 1600 that the island was uncultivated and uninhabited. Zinnanti [14] writes that “the same method of cultivation was later used in the fields and by streams by the populations of Spain, and on the island of Marettimo in the cultivable sites only, the remainder on behalf of the patron who rented it to loggers.”

Beekeeping on Marettimo was very important in the past, and it continues to be so today. Many authors in different times have written about this, including Orlandini [16] in 1605, Massa in 1709 [17], and Adorno in 1806 [18], who all described the island as rich both in thyme and honey bees.

Captain Smyth wrote a more detailed description of the island [15]:

“The westernmost of the Aegadian islands is Marettimo, formerly Hiera … is inhabited by fifty or sixty people, who cultivate the arable part, collect a considerable quantity of fine honey, and export fagots to Trapani.”

More than a century later, Dùran (1928), the Marettimo port official, wrote [19]:

“One of Marettimo's resources, in addition to that of honey and sponge fishing, carried out by Greek *trabaccoli* [sailing coaster], is that of fishing and salting sardines, collecting capers and excellent delicious mushrooms. The island is rich in excellent drinking water and many aromatic plants. Thyme, bay, myrtle, rue, hollyhock, rosemary, mastic, oak, oregano, alternate on its slopes, spontaneously generated by Mother Earth.”

Duran also wrote of the eventual abandonment of the landscape, “We take away from the current abandonment and give back to culture a good part of our island and we try to cultivate and sow the neglected and forgotten plots of land … The mountain was once more forested.” [19].

Francini and Messeri wrote in 1956 [20]:

“Agriculture is practiced on a very small scale, in a few small fields in the town or at the most important sources of water fountains… In spite of this, every stretch of land with a gentler slope bears traces of attempts at crops, and almost everywhere you can see more or less abandoned fields…Sumac was cultivated in the past, as well as manna ash, some vine reed and prickly pear fig; today these have been abandoned. Some trees of almost all fruits are found on the island: plums, cherry trees, peaches, apricots, apple trees, pear trees, and almond trees, with the tips of the branches all dried by the sea winds.”

Continuing in his description of the island flora, both cultivated and wild, he continues:

“There are about fifty olive trees; however, the oil is not made, but the olives are sold. There are some carob trees, which do quite well. Agave is abundant near the town; it is kept in a hedge and is used to make ropes to bind wood. Some figs are grown here and there, and there are many unsuccessful attempts to make them take. Some plots are vineyards, cultivated with low vineyards, as in Sicily. Broad beans are grown and, less, cauliflowers, spinach and beets. Barley and oats are grown among the cereals; wheat cultivation tends to be abandoned, given the very low yield. Shrubs provide all the fuel needed by the inhabitants.”

Of all three islands, Marettimo is the one that has recorded the greatest loss of diversity of fruit species.

The situation on the island of Favignana—the largest of the Aegadian islands—is different, where strips of agriculture persist, and despite widespread anthropization, it is still characterized by an agricultural landscape. Notably, the local cultivation of fruit orchards in the historic limestone quarries—similar in some ways to the cultivation of lemons in the quarries of Bagheria—is worthy of protection and conservation (Fig, 2 B). Favignana's human history, also due to its proximity to mainland Sicily, is certainly more articulated than that of the other two islands, as evidenced by the recent discovery of a Phoenician settlement (8th–7th century BCE). There is no evidence of agriculture from the Roman and Arab period and the subsequent ones, probably because this island was also involved in the depredation of the Barbary pirates and the Genoese.

Following the land concessions of the mid-1600s, the island passed to the Pallavicino family. Zinnanti (1912) states that only after 1700 and under the direct dominion of the Pallavicino barons, the wild trees were felled to begin cultivating the land, specifying that in 1700 “came to Favignana from Caltanissetta; certainly Antonio Li Volsi ... planted the fruit trees, and the island soon abounded in vegetables, cereals, corn, cotton and more. Then the Canino family came, planted vineyards, prickly pears, and other trees while growing cereals” [14]. However, Massa (1709) defines it as very fertile, and perhaps, as happened in Lampedusa, deforestation accelerated soil erosion and nutrient depletion [17]. This seems to be confirmed by the description of Amico Statella (1757–1760), who wrote:

“Favignana stands out for the fertility of the fields and the amount of rainwater, it is highly ideal for cultivation, as confirmed by Orlandino that the winds are favorable and increase the fertility of the soil. It nourishes the flocks with pasture, cultivates beehives, and then produces tasty cheese and honey, from which earnings are brought from the vicinity of Sicily up to Palermo. It presents abundant hunting of deer and rabbits.”

Monticelli Teodoro lived in Favignana from 1795 to 1801 and described beekeeping of Favignana. He describes the island in the 1800s as unhappy “without woods, without shrubland… in the summer a small amount of wheat and oats.” Yet, he also praised the work of men on the island, writing [21]:

“pushing on the sterile nature of these rocky cliffs, he dressed the island in some places with grapevines, provided some places with small gardens in the limestone quarries, and has introduced many vegetable gardens through digging wide wells and has scattered large quantities of prickly pear cacti and many local fig trees; he planted some very sweet grapevines, among which the *Apiana de ‘Latini*, also known as the *zibibbo* of Calabria or *moscadellone* so well appreciated by bees; many pomegranates, peaches, and other fruit trees. These improvements of the human art [of cultivation], helped by the climate and the nature of the island abundant with aromatic herbs, including saffron, thyme, mint, ivy, capers, chicory, the mastic tree, oak, and a kind of wild tea.”

In Levanzo, the agricultural landscape is still visible: the fields of the central part of the island are separated by dry-stone walls. The agrarian history of Levanzo is recent and begins with the introduction of vineyards by the noble Pallavicino family of the 17th Century. Historical references, in fact, only refer to the presence of bees, animals, and spontaneous wild vegetation. Orlandini (1605) writes: “Levanzo…is perhaps named by honey from holy Ptolemy…of which the bees make in those cliffs and steep slopes” [16]. Massa (1709) reported that Levanzo “abounds with timber” while Adorno (1806) praised its “pastures and verdant saplings” [17, 18]. Amico Statella (1757–1760) confirmed all of this, describing Levanzo as “of very high rocky cliffs, but nevertheless, it is abundant in pastures…also seen full of shrubs” [22].

Giuffrida (1982) dates the moment in which agriculture develops in Levanzo, writing that at the end of 1600 in Levanzo, “a vineyard of 96 thousand plants was built and a warehouse and a palm grove were built” [13]. Today, it is still possible to admire the remnants of the works built for the transformation of wine at this site (Fig. 2C). However, the abandonment of agricultural structures connected to cultivation are evident.

Based on what Captain Smyth writes (1824), Levanzo seems to have been colonized only recently: “Levanzo, the ancient Phorbantia; but it does not appear to have been settled or cultivated until the last century when a few houses were built in a valley between two ridges of hills, where a little grain and fruit is cultivated, and some sheep and goats are reared: great numbers of faggots are also made from the stunted woods, and sent to Marsala and Trapani for sale” [15]. Zinnati (1912) wrote: “The Island of Levanzo was the only one left for the benefit of the patron Pallavicina…planted his vineyard, which still exists to this day.” Today, only a few fruit species remain, including figs and olive trees, while the famous grape varieties have been lost.

### Study aims

These historical transformations of the landscape across the Aegadian islands have undoubtedly influenced how local people engage with the terrain today, covering the full spectrum of knowledge and use of cultivated and wild plants for food and the support of population health and well-being. Here, we report on an ethnobotanical survey undertaken on the circum-Sicilian Aegadian Isles of Italy, locally known as the “Isole Egadi.” Our research efforts were focused on the three largest and only inhabited isles of the five island chain, with interviews conducted in Marettimo, Favignana, and Levanzo. The central aim of this study was to document traditional ecological knowledge (TEK) concerning wild plants and fungi for food, health, and other household applications and to examine how present-day practices compare to historical reports.

## Methods

### Study site

The Aegadian Islands are a group of five small mountainous islands situated off of the northwest coast of Sicily in the Mediterranean Sea, with the three inhabited islands of Levanzo (38° 00' 02.4" N, 12° 19' 54.7" E), Favignana (37° 55' 53.7" N, 12° 19' 38.2" E), and Marettimo (37° 58' 28.3" N, 12° 03' 17.1" E) located roughly 13, 16, and 24 km west of Trapani. The two smaller islands of Formica (37° 59' 22.2" N, 12° 25' 29.3" E) and Maraone (37° 59' 24.9" N, 12° 24' 49.1" E) are positioned between Levanzo and the Sicilian coastline (Fig. 1). In all, the islands make up a landmass of roughly 37 km^2^ and, as of a 2017 census, are inhabited by 4292 people [23].

Marettimo Island is made up of four tectonic units, deriving from the deformations of Mesozoic substrate, consisting mainly of dolomites, marls, and limestones [24, 25], while Levanzo Island is characterized by carbonate and clastic-terrigenous deposits [26]. Favignana Island hosts a ridge of dolomite and limestone oriented North to South (Mt. Santa Caterina—312 m a.s.l.) and two large plain areas that indicate ancient surfaces of marine erosion; the eastern plain is covered by whitish sandstone deposits of the Lower Pleistocene, whereas the western one is characterized by Mesozoic-tertiary carbonate sequences, sometimes covered by more recent Tyrrhenian conglomeratic layers, Holocene aeolian sediments, and colluvial and eluvial deposits [27].

According to the classification of Rivas-Martinez (1995) [28], Favignana and Levanzo fall into the dry thermo-Mediterranean (Temperature mean annual 18 °C; Precipitation m. a. 500 mm), while the island of Marettimo is mainly included in the thermo-Mediterranean zone from dry to subhumid, but tending towards the subhumid Meso-Mediterranean over 400–550 m of altitude [29].

Belonging to the province of Trapani, the local government operates out of the Comune of Favignana. Some of the local landmarks include the “Grotta del Genovese” (Cave of the Genoese) cave site with Neolithic paintings and Palaeolithic graffiti on Levanzo, subterranean gardens in the remains of calcarenite limestone quarries of Favignana (Fig. 2B), and the “Case Romane” (Roman houses)—structural remains of a 150 BCE Roman garrison on Marettimo (Fig. 2A). More recently, the islands were known for their extensive tuna fisheries and processing plant on Favignana.

### Field study

A total of 48 in-depth interviews (typically lasting 2 to 3 h in duration) were conducted from May 2016 to July 2017 and October 2018 on the inhabited islands of Levanzo, Favignana, and Marettimo in the Aegadian Islands in Sicily, Italy (Figs. 1 and 2, Table 1). All interviews were conducted in Italian by CLQ, AS, ALR, LC, and SG. Study informants were recruited with the assistance of introductions by the Municipality of Favignana and snowball sampling methods. We aimed to target a mix of informants from various economic activities based on agriculture, maritime practices, and household work. Interviews were limited to native Aegadian islanders who have lived the majority of their life on the island. We included both individual and small group (2–3 informants) interviews in the study. In the case of group interviews, special care was taken to accurately document which informants spontaneously cited species information and when there was consensus or disagreement concerning local names and uses of the cited species. Prior informed consent was always verbally obtained before conducting interviews, and the ethical standards of the Society for Economic Botany and International Society of Ethnobiology were followed [30]. We employed semi-structured interview techniques to investigate traditional ecological knowledge concerning food, health, and economic activities on the islands.

**Table 1:** Summary of informant data

Island	Size (km^2^)	Max elevation (m.a.s.l.)	No. of men interviewed	Average age ± standard deviation	No. of women interviewed	Average age ± standard deviation	Total no. interviewed
Levanzo	5.82	278	8	65 ± 11	2	72 ± 26	10
Favignana	19.8	314	13	75 ± 10	14	78 ± 10	27
Marettimo	12.3	686	8	77 ± 9	3	75 ± 9	11
Total	37.9	--	29	74 ± 11	19	77 ± 11	48

For clarity, individual ‘*use citations*’ refers to each mention of a plant or fungus use by an informant. Use citation data encompasses part(s) used, mode(s) of preparation, mode(s) of application, intended use or purpose, and information concerning the folkloric value or relevance to local traditions. The term ‘*species use*’ refers to either a unique use citation by a single individual informant or a group of matching ‘*use citations*’ given by multiple informants. All use citation data were collated and organized in Microsoft Excel into species use groups for statistical analysis, described below.

Digital photographs and voucher specimens were taken for all available wild cited species. Vouchers were deposited in the Herbarium Mediterraneum Panormitanum (PAL) at the Università degli Studi di Palermo in Palermo, Italy and the Emory University Herbarium (GEO) in Atlanta, GA, USA. Specimens were digitized by GEO and have been made available on the SERNEC portal [31]. Herbarium specimens of all cited plant species were shipped to Emory under the USDA/APHIS permits PCIP-15-00957, PCIP-17-00110, and P526P-17-00635. Plant nomenclature follows the standard Italian flora [32, 33]. Fungal nomenclature follows MycoBank [34].

### Data analysis

#### Informant consensus factor

The categories selected for use in the informant consensus factor (*F*_*ic*_) analysis are provided in Table 2 and follow the system described by Trotter and Logan [35] and Heinrich et al. [36]. Each species use was added to the appropriate category before analysis using the following formula:

$$ {F}_{ic}=\frac{N_{uc}-{N}_t}{N_{uc}-1}, $$

**Table 2:** Division of ethnobotanical use reports by general categories for Informant Consensus Factor (*F*_*ic*_) analysis

	Examples of indications
General category of use	
Ethnoveterinary	Livestock feed, “healthy” fodder, forage, laxative, digestive aide, encourage weight gain for livestock
Food	Edible plants and fungi, cooked or raw ingredients, ingredient substitutes (e.g., coffee substitute), flavoring for liqueurs and grappa, snacks, seasoning, condiments, marmalade
Household	Games, cleaning tools, decorative (indoor and outdoor), fire-starter, home construction, window shade, baskets, agricultural tools, fencing, dyes, insect deterrent, ink, protectant, fiber source, pest repellent, firewood, saddle construction; environmental indicator of other species; acquired by trade with other islands or mainland; ritual applications; tools used to form the shape of food ingredients
Maritime	Fishing tools, fish poison, boat construction, fishing net dye
Nuisance	Pest plant (thorny, poisonous), problematic for people or livestock, crop allelopathy, cause of allergies, cause of contact dermatitis, skin irritant
Human medicinal uses	
Cardiological	Promote heart health
Dermatological	Lacerations and bleeding wounds, weak hair, burn wounds, abscesses, furuncles. skin and soft tissue infections, skin inflammation, hair loss, emollient for damaged skin
Gastrointestinal	Constipation, stomachache, colic, digestive aide, intestinal helminths, diarrhea, vermifuge
General health	To strengthen constitution, general wellness, refreshing beverage, “healthy” beverage or food (folk-functional food), calming agent, depurative, diabetes prevention or management, weaning from breast milk, anti-inflammatory beverage
Musculoskeletal/neurological	Arthritis, headache, fever, rheumatism, bruises, dislocations, edema
Optometric	Conjunctivitis
Oral health	Gingivitis, oral inflammations
Otolaryngological/respiratory	Sore throat, pharyngitis, cough, colds, antitussive
Urological	Urinary tract infection, kidney stones, diuretic

where *N*_*uc*_ is the total number of use citations in each category, and *N*_*t*_ is the number of species used in that category. High *F*_*ic*_ values (near 1.0) are obtained when one or a few species are reported to be used by a large proportion of informants for a particular category, whereas lower *F*_*ic*_ values indicate that informants disagree over which species to use (Table 3).

**Table 3:** Informant consensus factor analysis for plants and fungi

Category of use	Number of taxa (***N***_***t***_)	Number of use citations (***N***_***uc***_)	Informant consensus factor (***F***_***ic***_)
***General category of use***			
Ethnoveterinary	22	77	0.72
Food	56	378	0.85
Household	43	263	0.84
Maritime	9	35	0.76
Nuisance	6	29	0.82
***Human medicinal use***			
Cardiovascular	4	8	0.57
Dermatological	25	88	0.72
Gastrointestinal	13	94	0.87
General health	19	95	0.81
Musculoskeletal/ neurological	6	44	0.88
Optometric	2	2	--
Oral health	4	13	0.75
Otolaryngological/ respiratory	10	28	0.67
Urological	8	57	0.88
***Total***	***227***	***1211***	

#### Fidelity level

The fidelity level (FL) percent measure was used to identify the central role of each reported species [37], Additional File 1. The FL was defined as the ratio of between the total number of informants that independently cited a specific species use (*N*_*t*_) and the total number of informants (*N*) that cited the species for any use:

$$ FL=\left(\frac{N_t}{N}\right)\times 100 $$

This method’s primary limitation is that for species with only a few citations (≤ 3), the fidelity level may appear to be artificially high. Thus, species with three or fewer citations were excluded from this analysis.

#### Use-value citation index

The use-value (*UV*_*c*_) citation index, which is useful for evaluating the relative importance of each species based on its cited uses, was calculated for all species [38] (Additional File 1). Briefly, it is calculated as follows:

$$ {UV}_c=\frac{\sum {U}_{is}}{N}, $$

where *U*_*is*_ is the sum of the total number of all individual use citation reports concerning a given species, divided by the total number of informants (*N*).

## Results

**Fig. 3:**
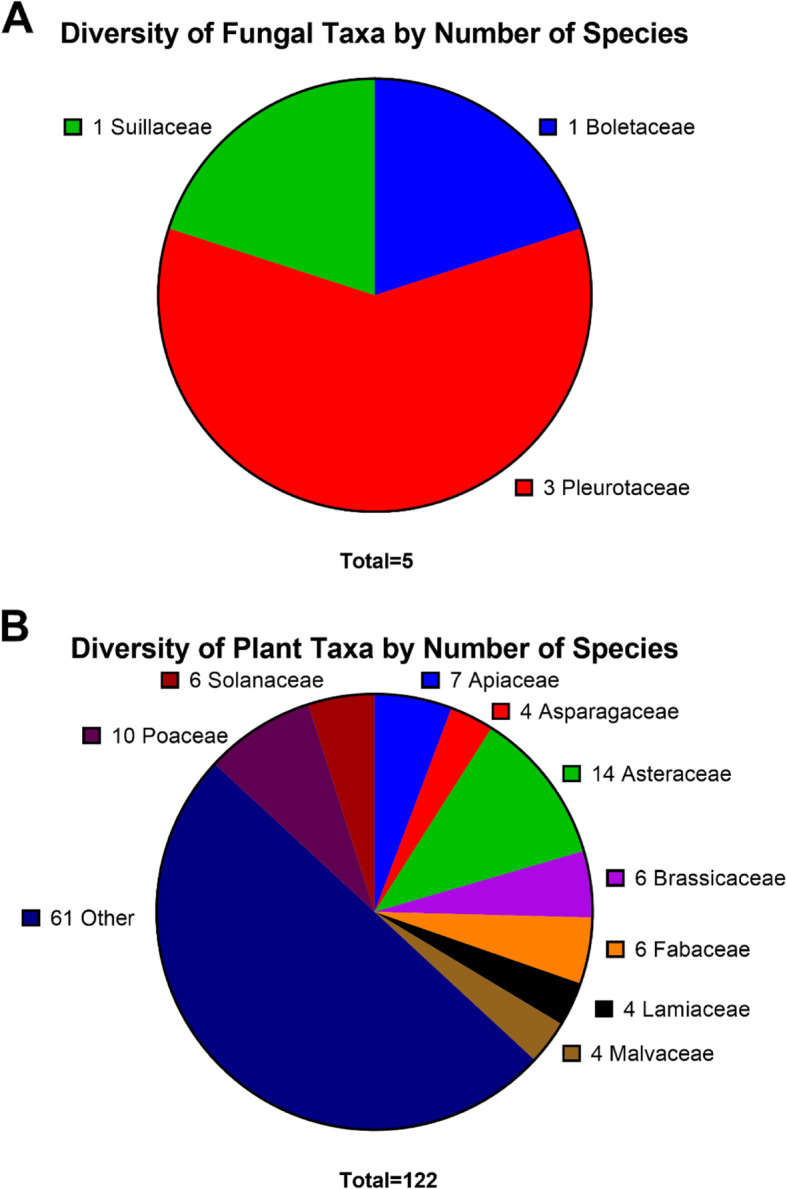
Taxonomic diversity of cited species divided by **A** fungi and **B** plants. Plant families with three or less species cited per family not listed in the figure (species grouped as “Other”), but can be viewed in Additional File 1

A total of 123 plant and five fungal taxa, representing 54 families (Fig. 3), were cited by the 48 study participants, divided across general categories (ethnoveterinary, food, household, maritime, or nuisance) of uses and human medicinal applications (cardiovascular, dermatological, gastrointestinal, general health, musculoskeletal/neurological, optometric, oral health, otolaryngological/respiratory, or urological) on the three inhabited islands of the Aegadian Islands.

Floristic investigation on these islands have reported a total of 492 taxa for Marettimo [29], 468 taxa for Levanzo [39, 40], and 570 taxa for Favignana [41]. Therefore, considering that most of the species are in common between the three islands, it can be extrapolated that the data we have collected on the ethnobotanical use of 123 taxa represent about a quarter of the total flora of the islands.

The informants' age ranged from 56 to 91, with a median age of 78 and a gender distribution of 60% male and 40% female (Table 1). Data on 1211 use citations were collected, representing a total of 355 distinct sets of collective knowledge regarding specific species (Additional File 1). With regards to the taxonomic distribution of species reported, the most represented plant families were Asteraceae (14 species), followed by Poaceae (10), Apiaceae (7), Brassicaceae (6), Fabacaeae (6), and Solanaceae (6), Fig. 3B. Here, we report the findings from our analysis of the ethnobotanical data reported by study participants.

### Informant consensus analysis

**Fig. 4:**
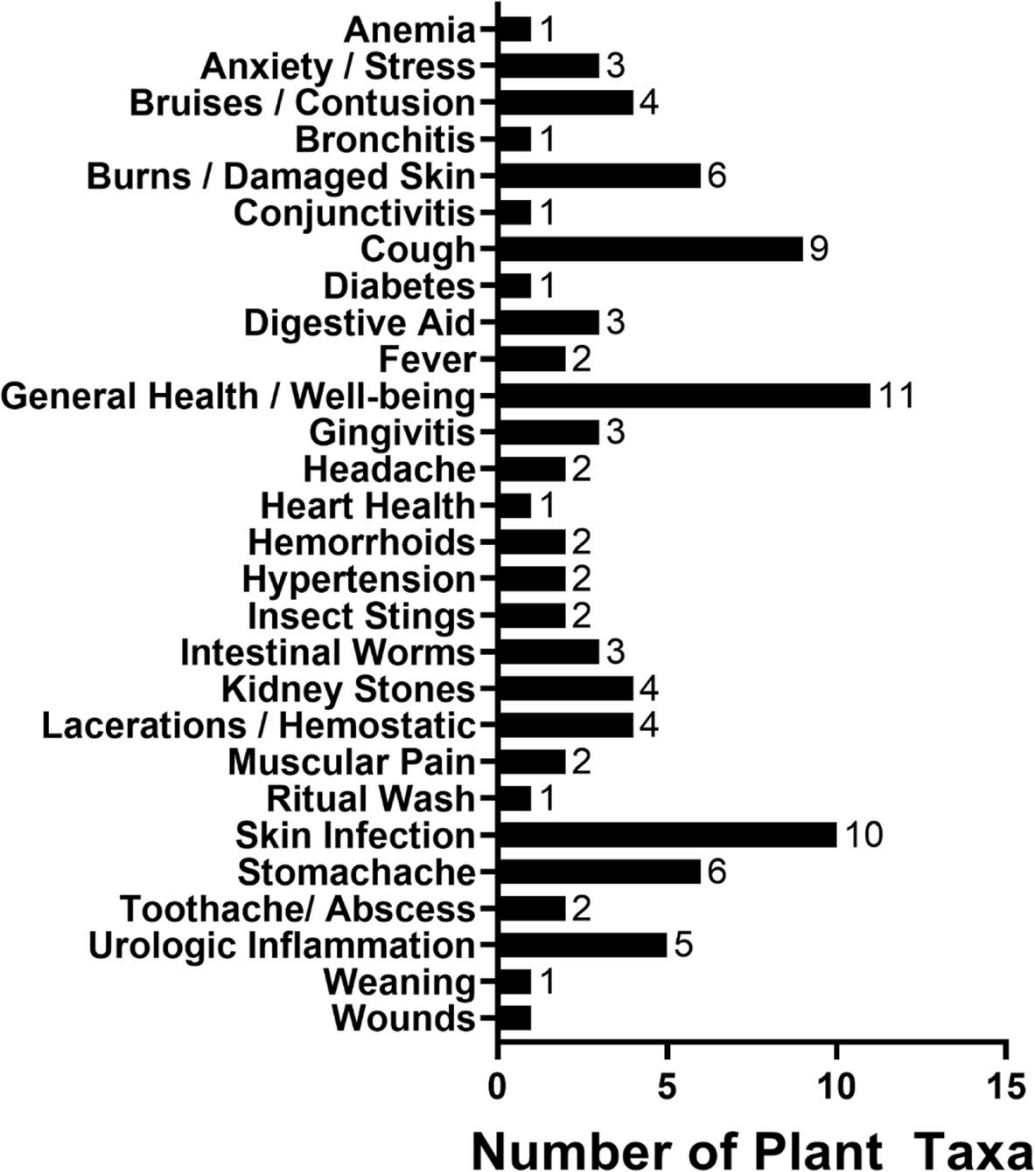
Number of medicinal plant taxa used to treat various reported ailments

Use reports for local plants and fungi were divided into five general categories and nine human medicine categories (Table 2). The greatest number of species (*N*_*t*_) reported for any category was for food (56 species), followed by household applications (43), and dermatological uses in human medicine (25), Table 3. The greatest number of use-citations (*N*_*uc*_) was also for the categories of food and household uses (378 and 263 use citations, respectively), but regarding human medicinal uses, the most use citations were reported for general health, gastrointestinal, and dermatological categories of use (95, 94, and 88, respectively), Table 3. Only four of the 14 categories had high levels of informant consensus (*F*_*ic*_ ≥ 0.85): musculoskeletal/neurological (*F*_*ic*_ = 0.88), urological (0.88), gastrointestinal (0.87), and food (0.85). The household category was close to this mark as well with an *F*_*ic*_ of 0.84. This relatively low level of consensus in the various specific use categories could be linked to differences in TEK from one island to another. When further broken down by specific uses within medical categories, the highest number of taxa were cited for use in general health and well-being (11 species), followed by skin infections (10), cough (9), burns/damaged skin (6), and stomachaches (6) (Fig. 4).

### Parts used and preparation methods

**Fig. 5:**
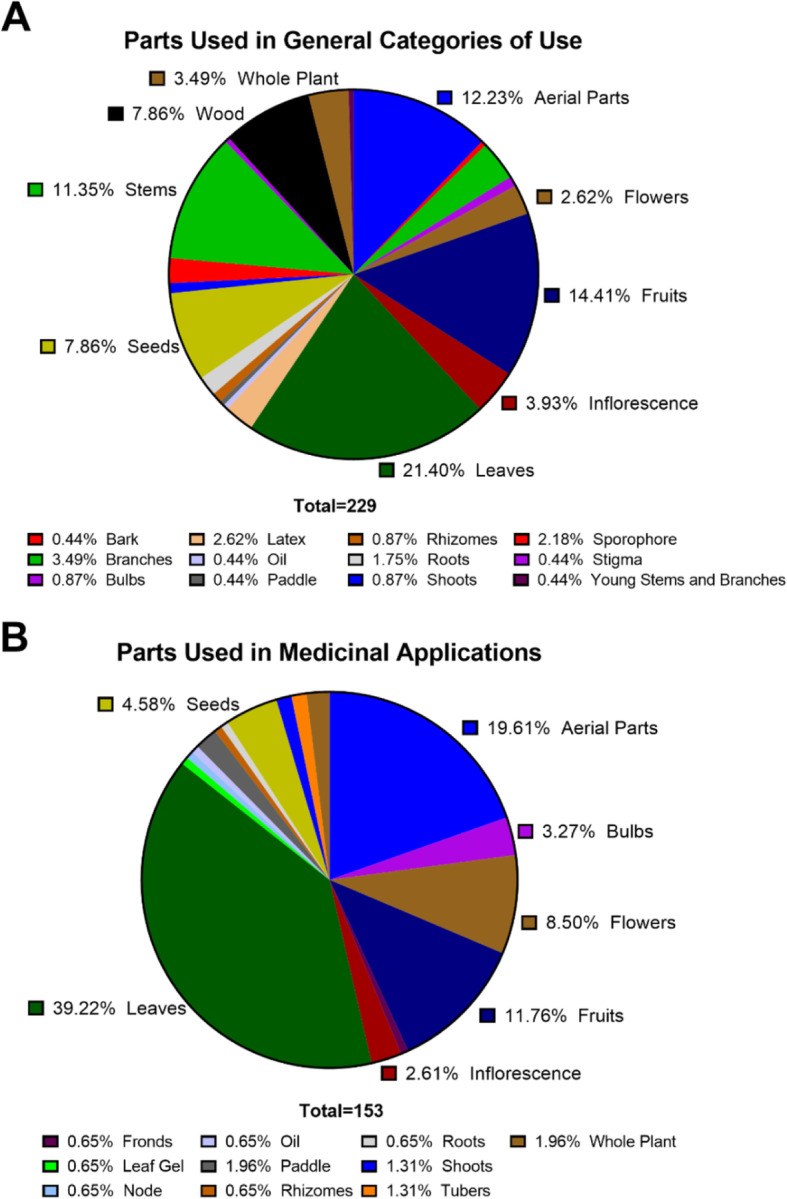
Parts of taxa used for **A** general categories and **B** human medicinal applications (see Table 2 for details on categories), represented as a percentage of reports within each grouping. For many taxa, different parts of the same species are used for different applications or the same parts of the species are used for distinct applications; these values are represented as discrete units in the figure

Regarding parts used in general categories of use, the most common parts were the leaves (21%), followed by fruits (14%), aerial parts (12%), and stems (11%) (Fig. 5A). Parts used in preparations for medicinal applications followed a somewhat similar trend, with the most common parts being leaves (39%), followed by aerial parts (20%), and fruits (12%) (Fig. 5B). The most common preparation method for medicinal plant parts was as a decoction (33%), followed by direct use (20%) and tisanes/infusions (13%) (Table 4).

**Table 4:** Preparation methods for medicinal plants documented in distinct preparation/use reports

Preparation method	Percentage of total reports
Boiled and plant part used	5.00%
Decoction	32.86%
Direct use	20.00%
Infusion/tisane	12.86%
Juice	0.71%
Maceration	2.14%
Mixed with bread	1.43%
Pestled with salt	6.43%
Poultice/cataplasm	11.43%
Powder	1.43%
Roasted over fire	0.71%
Syrup	5.00%

### Fidelity level analysis

Fidelity level (FL) analyses enable evaluation of the central role of a species in a study site; this is particularly useful when considering certain species with high numbers of use citations, but spread across different domains of utility. For example, *Pistacia lentiscus* L., Anacardiaceae was cited for use 18 times with a Use-value citation index (*UV*_*c*_) of 0.4 (Additional File 1). While it has cited uses in basket making and furniture making, its central role (FL = 89%) is as a source of firewood used in baking bread, and it is traded between islands specifically for this purpose. Other plants, on the other hand, have a wider range of utilities, with a high *UV*_*c*_ but a series of lower FL values. For example, *Agave americana* L. subsp. *americana*, Asparagaceae has a *UV*_*c*_ of 0.9, yet its highest FL value is 28% (for fiber making for stuffing chairs), followed by fibers for cordage (16%), and ashes for washing clothes (12%). In some cases, the final utility of the product may be quite similar, but its processing distinct. For example, *Sonchus oleraceus* (L.) L., Asteraceae, has a *UV*_*c*_ of 0.83, and it was reported as food prepared either by earing it raw in salads when leaves are young and tender (FL = 40%) or boiling older leaves to eat (58%).

### Use-value citation analysis

**Fig. 6:**
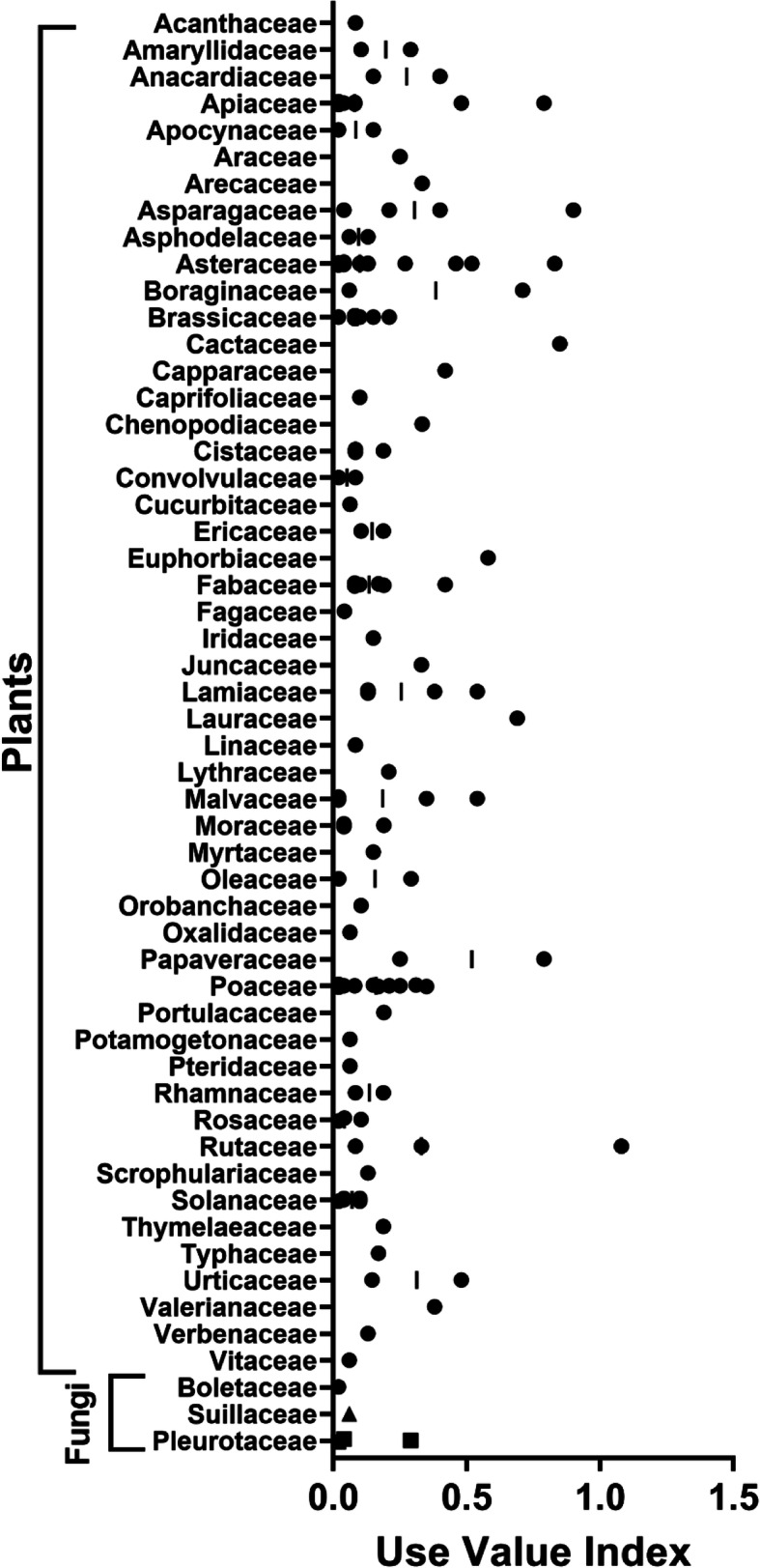
Use-value analyses by family

Use-value citation indices were determined for all cited species and are reported in Additional File 1. This analysis enables determination of the overall rank of species by informants, allowing for a comparative analysis at the rank of individual species, but also at the taxonomic rank of plant family (Fig. 6). The species with the highest use-value indices were *Ruta chalepensis* L., Rutaceae (*UV*_*c*_ = 1.08), *Opuntia ficus-indica* (L.) Mill., Cactaceae (0.85), *Sonchus oleraceus*, Asteraceae (0.83), *Glaucium flavum* Crantz, Papaveraceae (0.79), *Borago officinalis* L., Boraginaceae (0.71), and *Laurus nobilis* L., Lauraceae (0.69). Notably, each of these highest ranked species are distributed across different families. Comparison of use-value indices at the family level revealed that the Cactaceae, Papaveraceae, and Rutaceae had the highest average use-values, though this analysis is limited by disparate numbers of representative taxa within each family group.

## Discussion

### Historic cultivars

In Marettimo, there are still fruit trees in the small orchards of the town, even if the names of the old varieties have been lost. There are some plums grafted on almond trees: one cultivar with purple fruit, more watery, one variety always with purple fruit, and two plants with white fruit. Unfortunately, in Marettimo, two cultivars of peach have disappeared, a cultivar of apricot (dialectal name: *pircoca*), a cultivar of nectarine (dialectal name: sbergi)—probably the same still present in Favignana and the “azzeruoli,” which were present almost exclusively in the old orchards. These were probably grown for the fruit or as a rootstock for pome fruits. There is a cultivar of “pirazzolo” pear and black mulberries “ceusu”. At the historic site of the Roman Houses, there are figs, two plum trees (one with sour purple fruit and one with a sweet seed, “azzeruoli”), and some almond trees. Around the village, there are still a few pomegranates, pear trees, figs, mulberries, and some almond trees.

Due to the isolation that characterized this island and the rapid decline in agricultural activities, these few fruit trees still play an important role for local inhabitants. Likewise, fruit trees also play an essential role as a food source for birds. The delicious fruits of the black mulberry tree, for example, attract numerous small birds, as is evident to those who stay for a while in its shade.

Unfortunately, even if the trees are still present, memory of the local dialect names with which they were once recognized has been lost. Every effort should be made to recover the names of the varieties and use them from the old inhabitants, in Marettimo, as elsewhere, before this heritage disappears together with the men and women who created and maintained it.

### Current trends in traditional ecological knowledge

Current TEK trends reflect an adoption of introduced species along with other native flora in the medical traditions of the island. Here, we highlight some of the species most commonly cited by Aegadian islanders and examine their historic and contemporary uses.

#### *Agave americana* L. subsp. *americana*, Asparagaceae

The century plant is of North American origin, but is now naturalized in Sicily, where if planted in narrow rows can be used to create a reinforced fencing wall to delineate property boundaries. This use is made possible thanks to the presence of aculeate leaves with sharp terminal thorns that discourage the intrusion of grazing cattle [42]. For the Trapani community, an infusion of leaves was administered as a purgative to animals (horses, cattle, pigs) that were constipated. A poultice of the leaves and rhizomes was used to mature boils on the skin. In ancient times, these were planted at the edge of dirt paths between farms (trazzere), or to delimit property borders of farms [43]. In Mazara del Vallo, for the vigil of the feast of the Assumption, torches were made with the dried floral scape emptied at the top of agave, locally known as *“*zabbara,” in which a rag with oil was placed [44]. On the Sicilian island of Pantelleria, situated near the Tunisian coast, the plant is also referred to as zabbara, and the dried inflorescence was historically used as a mast for small sailboats and for the construction of gates and fencing [9].

A very resistant fiber was obtained from the leaves, which, when properly woven, provided a cord used mainly as a tie to affix cultivated plants to support poles. Today, with the advent of synthetic fibers, this particular us has almost wholly disappeared. Until recently, the leaves were also widely used by artisans to make the stuffing of rustic chairs. Today, this practice is only found among those who still strive to remake period furniture [42]. In eastern Sicily, this species was topically applied on bruises, and this utility was also reported by a few informants in the present study. The leaves were removed and crushed to obtain a very thick mush to be applied for a few days until the hematoma disappeared [42, 45]. However, local healers advise not to prolong this application, as long periods of treatment could yield caustic effects [46].

#### *Artemisia arborescens* (Vaill.) L., Asteraceae

Tree wormwood is a plant whose distribution includes the southern Atlantic and Mediterranean coasts [47]. All the parts of the plant, but especially the leaves, have a bitter principle that makes it valuable as a tonic remedy that was used in stomach diseases, in intermittent fevers, and in scrofula (symptoms of which included swelling of lymph nodes in the neck). In ancient times, within the Sicilian territory, it was generally used to carry out aromatic baths and to wash chronic wounds [48]. The fresh parts of the plant were topically applied to treat acne and skin pustules; decoctions were used for internal veterinary applications only [45]. In folk medicine, this wormwood is used as an antimalarial and balsamic. The antimalarial drug is prepared by shredding the leaves, adding water to the mixture and boiling it until the mixture is reduced to half of the original volume. The decoction thus obtained, which according to the locals, is more effective than quinine, should be filtered with a cloth and administered to the patient three times a day [46].To have a soothing effect, a decoction was made of the new sprouts and administered to patients suffering from respiratory tract diseases [46]. Known in the Trapani area as “erva janca” (white herb), a leaf decoction was used as an anti-inflammatory and antipyretic, especially for veterinary use. Leaves were added to the cattle diet to reactivate rumination. In ancient times, to treat asthma, shepherds used to smoke dry leaves with their pipes [43].

According to the local tradition of the territory of Enna, it was necessary to intervene with a magical therapeutic practice to eliminate the parasites that would occur following panic or sudden anger events. The ritual was called “ciarmari i virmi” (calming the worms) or “nsurtiri i virmi”, practiced by some elderly custodians (known as “ciarmaturi”) of the rite to which it was handed down orally from the previous generation. For ciarmari i virmi, the affected child is made to smell aerial parts and leaves of the ground plant, and then he is placed in a supine position so that the ciarmaturi can practice, with his left hand greased with holy oil, circular massaging movements on the belly, reciting a formula that would have the power to eliminate parasites [49]. Similar uses of this species against helminthiasis have been reported in other areas of Sicily [50].

#### *Ruta chalepensis* L., Rutaceae

This species of rue has a strict Mediterranean distribution [47]. Throughout the Sicilian territory, the leaves were used as stimulants and irritants both as to activate menstruation and as a vermifuge. The vermifugal properties of rue are well known in the region [50]; there is a local belief that in order to make children expel pinworms, it is sufficient to have children deeply smell a bundle of this herb [43, 46, 51]. It also acts as a rubefacient when topically applied to the skin. However, it is a plant with a certain degree of toxicity. Ingestion of seeds (in excessive doses) causes severe gastrointestinal symptoms, diarrhea, depression, convulsions, and shock with slow heart rate [52]. Thus, rue was used with due precautions by women as it could stimulate inflammation and excessive bleeding in the uterus [48].

Historically in the territory of Trapani, an oleolite preparation of rue was created by macerating the leaves and flowering tops of the plant in olive oil. This was used by traditional healers (known as “mago”, “u spiritaru”, or a “mara") in spiritual-magical healing rites; the oleolite was used to anoint the stomach (rubbed on the umbilicus) of children suffering from helminthic infection while reciting a prayer [43].

In western Sicily, there are various medicinal applications of rue. A decoction is prepared from the aerial parts, and when drunk in small quantities a few days prior to menstruation, is reported to reduce the problems of dysmenorrhea. A tincture of the green or dried plant material is made by soaking in ethanol for a couple of months, and then topically applied through rubbing the resulting liquid on body parts affected by muscle pains, strains and sprains [53]. In the Nebrodi territory, on the other hand, an alcoholic tincture for use in treating rheumatic pains is prepared by macerating rue leaves with orange and mustard seeds in ethanol [45].

In eastern Sicily, leaf infusions were used against painful menstruation, for enemas, against worms, for baths against sciatica, and against heartburn. The crushed and heated leaves were also topically applied on bruises or to treat rheumatism. The whole fresh plant was also used to ward off insects and mice due to the bad smell it emanates [54].

#### *Glaucium flavum* Crantz, Papaveraceae

The yellow horned, or sea poppy is a species with an area centered on the Mediterranean coasts, but with extensions to the north and east. Inland, its distribution range is between Scandinavia and the Iberian Peninsula [47].

In his work on the Sicilian medicinal flora, Calcara (1851) identifies the plant with the term “caulu marinu”; this data highlights how, this vernacular name is not only reserved for the district of the Aegadian islands but had a greater distribution at regional level [48]. Calcara further reported that the plant has an unpleasant odor and contains a narcotic principle. The leaves were used as a purgative, but internal use was carried out with caution due to the suspicious action it has on the nervous system; it was thus used more commonly externally for the treatment of dermatitis [48]. In ancient times, it was used and recommended to treat jaundice, obstructions of the liver and spleen, dropsy, and intermittent fevers [55]. In the present study, we found that the most common use reported by Aegadian islanders and unique to these islands was topically for the treatment of hematomas.

#### *Pleurotus eryngii* (DC.) Quél. s.l., Pleurotaceae

The two fungal taxa highlighted in the present study have always represented a delicious food and a coveted and appreciated dish for the whole territory of Trapani, but also in the rest of Sicily. *Pleurotus eryngii* (DC.) Quél. var. *eryngii* and *Pleurotus eryngii* var. *ferulae* (Lanzi) Sacc. are saprotrophic fungi that live on rotting debris of certain members of the Apiaceae family: *Eryngium campestre* L. and *Ferula communis* L. subsp. *communis*, respectively. They are found mainly in Mediterranean grasslands disturbed by grazing, which clearly select for the plants involved in the fructification of the fungal sporophores. The presence of these species, quite frequent throughout the Sicilian territory, is therefore indicative of the anthropic disturbance. This factor plays a significant role in the distribution of plant communities to the detriment of potential vegetation, in Sicily by now rarefied and limited to a few areas.

### Comparison to past use reports

Many ethnobotanical uses of plants have now disappeared from the popular tradition due to changes that have occurred in the local economy, that is now mainly based on tourism, but also due to a greater use of pharmaceutical medications. A subset of 54 plant species (about 44% of the total) had in the past also different applications (mainly medicinal) with respect to current citations [56–59]. More than 70% of these uses concerned general health and specific remedies for dermatological, gastrointestinal, and urological problems (Additional File 1).

The literature on the traditional use of plants in the Aegadian Islands is very limited and consists of three rather old scientific articles [56–58] and one popular science book [59]. These studies adopted different criteria than current ethnobotanical methodologies (e.g., the number of informants was not reported), allowing only qualitative comparisons between previous and current data. In addition, past ethnobotanical investigations were mainly focused on medicinal and food plants, while a few data about other uses (household, ethnoveterinary, etc.) were considered. In contrast, our survey reports all ethnobiological uses of local taxa cited by informants (Additional File 1), for example the use of *Arisarum vulgare* Targ. Tozz. subsp. *vulgare*, Araceae as environmental indicator and as pig fodder, the decorative/religious role of *Matthiola incana* (L.) W.T. Aiton subsp. *incana*, Brassicaceae and *Myrtus communis* L., Myrtaceae*,* and the uses of *Juncus acutus* L. subsp. *acutus*, Juncaceae and *Ampelodesmos mauritanicus* (Poir.) T. Durand & Schinz, Poaceae to weave (along with young olive and pomegranate branches) ferret carriers for hunting. Maritime uses of wild plants related to fishing and seafaring were also recorded, including *Euphorbia dendroides* L., Euphorbiaceae (to catch fish), *Juncus acutus* (to weave fish traps), *Salvia rosmarinus* Schleid., Lamiaceae (to dye nets) and *Ampelodesmos mauritanicus* (to make rope). As a general trend in the Mediterranean area, these uses are the most rapidly disappearing in favor of tourism, and therefore their heritage value is maximal [9, 60]. In such contexts, small islands play an important role as biocultural refugia to preserve traditional uses and techniques of these marginal habitats. As for the uses that have disappeared for decades, in some cases, they concern toxic and poisonous plants, such as *Rhus coriaria* L., Anacardiaceae, *Nerium oleander* L. subsp. *oleander*, Apocynaceae, and *Mandragora autumnalis* Bertol., Solanaceae.

Older people remember very well that young stems and branches of Sicilian sumac (*R. coriaria*) in the past were sold as a dye, and widely used to stain fishing nets. This species was cultivated in the past both in minor isles, such as Lampedusa [61], and in Sicily (e.g., in the area of Bronte where in the 1930–1940s wild plants were harvested, processed, and traded in the tanning industry) [42]. However, a medicinal use of this species was also previously reported for the Aegadian Islands by Bonomo and Trapani (1974), who cited the use of bark, leaves, and fruit in the form of decoction, water extract, or as a powder, against fever and as a hemostatic [57]. Today, these specific uses have been lost, and the species is exclusively known for being an important melliferous plant.

**Fig. 7:**
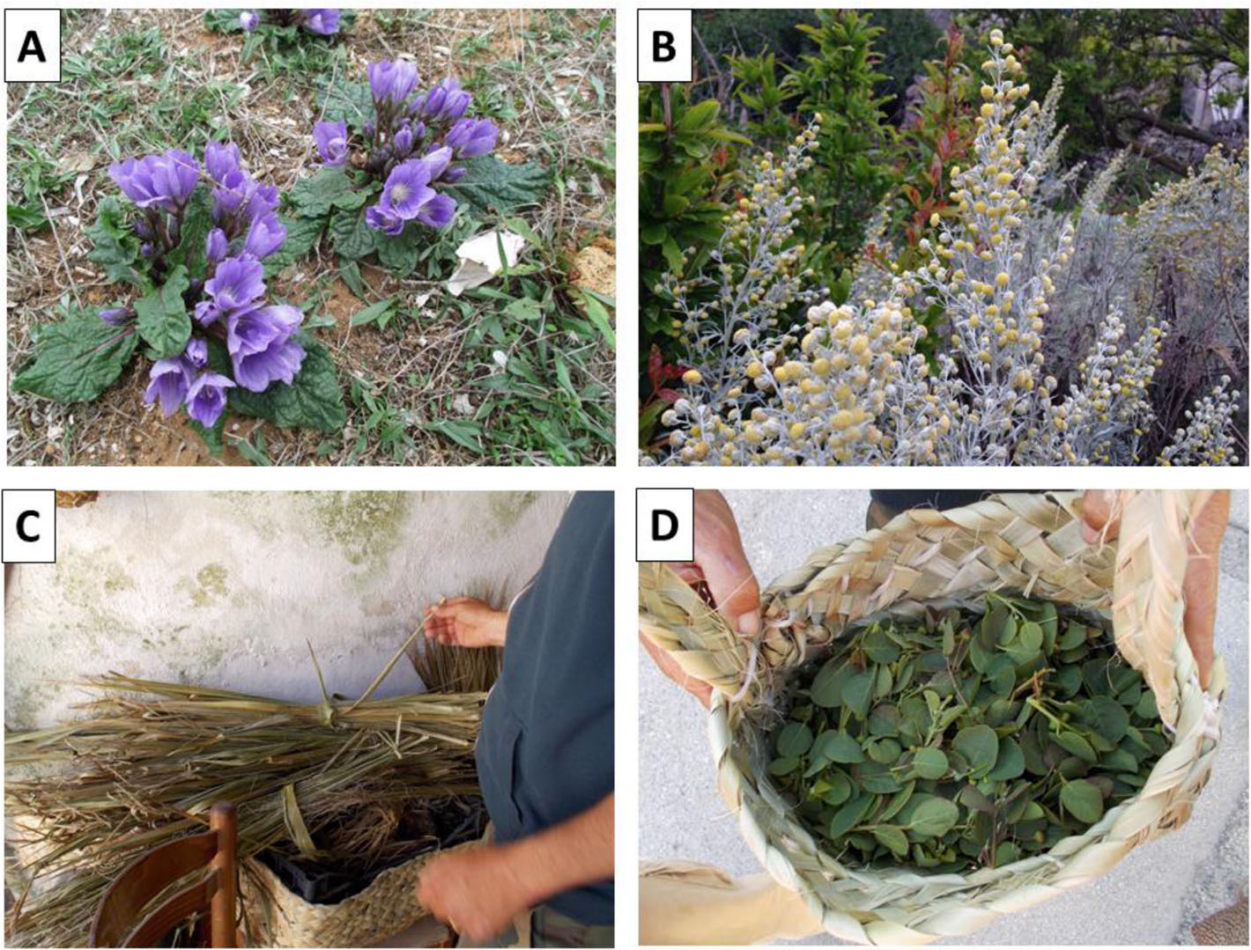
Example of species with historic uses on the Aegadian islands. **A** *Mandragora autumnalis*, a poisonous plant. **B** *Artemisia arborescens*, locally known as *vagnu*. **C** *Washingtonia* sp. leaves used to make baskets. **D** Handcrafted basket, made with *Washingtonia* leaves, for the collection of capers

In the past, fresh flowers of oleander (*N. oleander*) were placed on windowsills to deter insects from entering the house, while the leaves and bark of this species were curiously used to resolve sneezing [57]. Currently, the inhabitants of the islands know that mandrake (*M. autumnalis*) (Fig. 7A) is a poisonous plant, although often confused with *Borago officinalis* [62], but until the end of the 1990s, the fruit of this species was employed for medicinal purposes, such as in the treatment of articular pain [56, 58]. An even earlier medicinal use of the mandrake root is as antispasmodic to treat gastrointestinal problems [57]. Medicinal uses of mandrake were referred also from rural communities of Trapani Mountains (Western Sicily), where a poultice prepared with leaves and fruits, macerated in vinegar, was used against pain [43].

Some species of Asteraceae, currently recognized as wild vegetables, showed different medicinal uses over time, such as *Hyoseris radiata* L. (dialectal name: “cardedda i serpi”) that in the past was topically employed for skin disease [57, 58], while today, it is administered as a decoction for its litholytic activity against kidney stones. On the contrary, the use of *Centaurea calcitrapa* L. (dialectal name: spinaprocchia) as famine food, as well as to treat fever and as a diuretic and eupeptic remedy, is no longer adopted [57, 59].

The species *Artemisia arborescens* (dialectal names: “vagnu” and “erva janca”) (Fig. 7B) still plays an important role for its dermatological properties linked to a ritual context, being used to prepare a water infusion for the bathing of an infant or newborn, but it is also considered an antiseptic and cosmetic perfume for the infant’s skin (FL = 68%, Additional File 1). However, in the past, the plant was employed by inhabitants of the islands also for internal use as a digestive, vermifuge, cholagogue, and tonic [57]. Different medicinal uses of this species have been referred in many areas of Sicily, where the plant had a reputation as a panacea and was mainly used as vermifuge, antimalarial, and antipyretic [44, 63]. Also, a medicinal use of the decoction for veterinary applications has been reported in the rural area of the Trapani Mountains [43].

In addition to different plants quoted in the past as vermifuge, such as *Artemisia arborescens*, *Ruta chalepensis*, *Allium sativum* L., Amaryllidaceae, *Crithmum maritimum* L., Apiaceae, *Anagyris foetida* L., Fabaceae, and *Portulaca oleracea* L., Portulacaceae (Additional File 1), also red algae were used for this purpose by Aegadian islanders. An aqueous extract made with different red algae collectively called “semenza di vermi”, gathered on the rocks in the intertidal zone, was widely used in the past against worm infections in children, and it was even marketed out of the islands until the post-war period. Recently, the anthelminthic properties of these red algae, including the following members of the Rhodomelaceae family: *Palisada tenerrima* (Cremades) D.Serio, M.Cormaci, G.Furnari & F.Boisset, *Laurencia intricata* J.V.Lamouroux, and *Laurencia* spp., have been confirmed *in vitro* on gastrointestinal nematodes of donkeys [64]. Other data reported similar use of another Rhodophyta, *Corallina officinalis* L., Corallinaceae, called with a similar name (“simenza rê viermi”) in the Ragusa province [50].

Unripe buds of Capparis orientalis Veill., Capparaceae, locally known as “chiappara”, widely growing all over the islands, are commonly collected and conserved under salt, as shown by a FL of 100%. In addition, some people also weave palm leaves (*Washingtonia* sp., Arecaceae) to make special baskets used in harvesting (Fig. 7C, D), while some inhabitants sell jars of salted capers to tourists in the port area of Favignana. In our investigation of current TEK, capers (*C. orientalis*) were only cited for their food uses (lactofermented in brine), but in the past literature on the region, these were reported for a variety of medicinal applications, including as a depurative, gastrointestinal tonic, and anti-arthritic. In addition they were used to prepare a very bitter decoction quoted for its emetic and antimalarial activities in the past [56]. Similar medicinal properties were attributed to this species also in Iranian traditional medicine, where roots, fruit, and bark were used as antimalarial agent [65]. On the other hand, also the root bark of a related species, *Capparis leucophylla* DC, known as “Capparis Cortex Radicis” in the old Persian Pharmacopoeia, was indicated for the treatment of intermittent fever [66].

Other medicinal uses that have disappeared concern the seeds of different species: those of *Raphanus raphanistrum* L. subsp. *raphanistrum*, Brassicaceae were quoted as anti-rheumatic [57], while today, only the young leaves and tender stems of the plant are collected and eaten; the seeds of *Juncus acutus* were employed as diuretic [56, 58].

*Verbascum sinuatum* L., Scrophulariaceae is widely used to make brooms (FL = 100%)*,* a common use also reported in other areas of Sicily [43], but in the past, it was also used for medicinal purposes in the treatment of gastrointestinal, urological, and respiratory problems [57]. *Clinopodium nepeta* (L.) Kuntze subsp. *nepeta*, Lamiaceae, on the other hand, was cited by our informants for both its food and medicinal uses for stomachache and as a digestive stimulant, but in the historical reports [56, 58, 59], it was widely reported also for treating respiratory ailments. Similarly, medicinal uses of the flowers of *Erica multiflora* L. subsp. multiflora, Eriaceae, were referred to the past, namely as diuretic, depurative, sedative, and antirheumatic [57], while only the use as firewood (FL = 56%) has been preserved up to the present. The use as a fire starter for wood stoves has been described also for the related species *Erica arborea* L. at Pantelleria Island [9]. Likewise, although the use of wood for various applications was historically important in the Aegadian islands [67], this use did not emerge during our interviews. However, it is known from the literature that *Fraxinus ornus* L., Oleaceae and *Morus alba* L., Moraceae, were important in in Marettimo [68] and *Melia azedarach* L., Meliaceae, in Favignana [67].

In general, comparison with other data of the Sicily ethnobotany showed that similar uses of plants can be found more frequently in other small islands, characterized by a similar economy [9, 69], and in neighboring areas, such as the territory of Trapani [43].

Overall, the set of our data on the Aegadian Islands allowed to collect information on 127 taxa, against the 151 taxa reported in 1974 [57], forty-seven years earlier, showing a good conservation of the TEK. Furthermore, data concerning the maritime uses of wild plants and the techniques related to fishing and seafaring, which are in danger of being lost quickly [9, 60], remain in the memories of the eldest subset of the population. Hence, our data show that Aegadian Islands still represent important biorefugia for the conservation of the intangible cultural heritage of Mediterranean people.

## Conclusions

In Sicily, the loss of traditional ecological knowledge (TEK) has been reported for the domain of wild plants used as medicines [70]. However, the plant uses for fishery and agroecosystem management have also largely been lost in the socioeconomic changes that followed the Second World War. Regarding the use of plants in fishing activities along the Western Mediterranean Italian coasts, a decline of traditional fishery knowledge has been documented [60]. This has occurred in particular in small islands, where today the economy is mainly based on tourism [9, 60]. On the other hand, ethnobotanical studies have shown that in Sicily, a high number of wild taxa are still utilized as vegetables [71, 72], especially those at the interface between food and medicine [73].

In the Aegadian Islands, we had a unique opportunity to examine a group of populations living in isolated locations, to investigate surviving TEK on wild plants and fungi for food, health, and other economic and household applications, in both practice and memory of native people. Here we found that many plant-based traditions have disappeared from daily practice, especially those related to traditional fishing and healthcare practices, and to a lesser extent also several uses of wild plants for human/animal medication. In these isolated locations, the adaptive cultural identity of native islanders has been built over time based on self-reliance in procuring food and medicines for themselves and their livestock under difficult conditions (i.e., scarcity of fresh water, high temperatures and insolation, and strong wind). In this context, many specialized skills tailored to survival contributed to form the core of the TEK.

In our survey, we found that a large number of taxa are collected and used as food, such as the tender aerial portions of many wild species belonging to Asteraceae and Brassicaceae, or to *Fedia graciliflora* Fisch. & C.A.Mey. belonging to Valerianaceae (according to Bartolucci et al., 2018 [32]). Many wild taxa are also used as seasoning, not only *Capparis orientalis,* but also *Foeniculum vulgare* Mill. subsp. *vulgare*, Apiaceae, to prepare different local specialty dishes, such as “pasta con le sarde” and “polpette di finocchietto”, and in addition *Clinopodium nepeta* to flavor fish, especially tuna. We also observed that some traditions concerning medicinal uses of plants have been well preserved, such as *Glaucium flavum* for the treatment of hematomas, *Ruta chalepensis* for general health and gastrointestinal problems, and the dermatological ritual uses of *Artemisia arborescens*, species that still play an important role for the cultural identity of the Aegadian islanders.

TEK is handed down through generations by cultural transmission, representing an important resource for community resilience and cohesion in response to environmental and economic changes [8, 74]. However, most of this knowledge only remains in the memories of eldest Aegadians, who have retained a strong attachment with their own cultural traditions and who carried out themselves the typical activities that have been the source of livelihood for centuries.

The places with people who transmit traditional ecological knowledge and practices are considered biocultural refugia, providing genetic and cultural reservoirs for a wide array of species co-evolved with humans and a shelter for biodiversity and ecosystems [10, 75]. The decline in the regular use of TEK in the communities of small Mediterranean islands threatens their security as biocultural refugia. Consequently, documenting this knowledge before it disappears from oral history has a pivotal role in reducing loss of TEK and biocultural diversity [74].

## Supplementary Information

**Additional file 1.** Ethnobiological uses of local taxa. Data table reporting the specific uses of local taxa documented in the present work and comparison to prior publications in the region. (.pdf file format).

## Data Availability

All data generated or analyzed during this study are included in this published article. Specimens collected during this study deposited at Emory University Herbarium are digitally available on the SERNEC portal at http://sernecportal.org/portal/.

## Abbreviations

GEO: Emory University Herbarium
PAL: University of Palermo Herbarium
TEK: Traditional ecological knowledge

## References

[1] IUCN (2008). The Mediterranean: a biodiversity hotspot under threat: IUCN Species Survival Commission.

[2] Mustafa B, Hajdari A, Krasniqi F, Hoxha E, Ademi H, Quave CL, Pieroni A (2012). Medical ethnobotany of the Albanian Alps in Kosovo. J Ethnobiol Ethnomed.

[3] Mustafa B, Hajdari A, Pieroni A, Pulaj B, Koro X, Quave CL (2015). A cross-cultural comparison of folk plant uses among Albanians, Bosniaks. Gorani and Turks living in south Kosovo. J Ethnobiol Ethnomed.

[4] Pieroni A, Nebel S, Quave C, Münz H, Heinrich M (2002). Ethnopharmacology of liakra: traditional weedy vegetables of the Arbëreshë of the Vulture area in southern Italy. J Ethnopharmacol.

[5] Pieroni A, Quave CL (2005). Traditional pharmacopoeias and medicines among Albanians and Italians in southern Italy: a comparison. J Ethnopharmacol.

[6] Pieroni A, Quave CL, Santoro RF (2004). Folk pharmaceutical knowledge in the territory of the Dolomiti Lucane, inland southern Italy. J Ethnopharmacol.

[7] Pieroni A, Sõukand R, Quave CL, Hajdari A, Mustafa B (2017). Traditional food uses of wild plants among the Gorani of south Kosovo. Appetite.

[8] Quave CL, Pieroni A (2015). A reservoir of ethnobotanical knowledge informs resilient food security and health strategies in the Balkans. Nature Plants.

[9] Quave CL, Saitta A (2016). Forty-five years later: the shifting dynamic of traditional ecological knowledge on Pantelleria Island. Italy. Economic Botany.

[10] Barthel S, Crumley C, Svedin U (2013). Bio-cultural refugia—safeguarding diversity of practices for food security and biodiversity. Global Environmental Change.

[11] La Mantia T, Sottile F, Carimi F (2013). La frutticoltura delle isole circumsiciliane. Frutti dimenticati e biodiversità recuperata Il germoplasma frutticolo e viticolo delle agricolture tradizionali italiane Casi studio: Isole della Sicilia, Lombardia Quaderni Natura e Biodiversità. Università degli Studi di Palermo.

[12] Sottile F, Barone E, La Mantia T: Cenni storici sulla frutticoltura delle isole della Sicilia. In: Frutti dimenticati e biodiversità recuperata Il germoplasma frutticolo e viticolo delle agricolture tradizionali italiane Casi studio: Isole della Sicilia, Lombardia Quaderni Natura e Biodiversità. Università degli Studi di Palermo, Regione Siciliana: ISPRA, ARPA Emilia Romagna, ERSAF; 2013: 12-16.

[13] Giuffrida R (1982). I Pallavicino e le isole Egadi. La Fardelliana.

[14] Zinnanti M. Cenni storici delle isole Egadi. Int Dell’ed del 1912 a cura dell’Associazione Marettimo, Trapani. 1912:43.

[15] Smyth WH: Memoir descriptive of the resources, inhabitants and hydrography of the Sicily and its islands, interspersed with antiquarian and other notices. In: La Sicilia e le sue isole Italian translation by GD Catinella e G De Franchis, Ed Giada. Palermo; 1824: 358.

[16] Orlandini L: Trapani succintamente descritto del Canonico Leonardo Orlandini. Trascrizione a cura di G. Lipari. http://www.trapaninostra.it/libri/Gino_Lipari/Trapani_Succintamente_Descritto/Trapani_Succintamente_Descritto.html. 1605.

[17] Massa GA (1709). La Sicilia in Prospettiva. Parte Seconda. Cioè Le Citta, Castella, Terre e Luoghi esistenti e non esistenti in Sicilia, la Topografia Littorale, li Scogli, Isole e penisole intorno ad essa. Esposti in Veduta Da Un Religioso Della Compagnia Di Gesù.

[18] Adorno D (1806). Descrizione geografica dell’Isola di Sicilia e dell’altre suen adiacenti.

[19] Dùran PE (1928). Una perla in fondo al mare, sintesi storica-politica-sociale dell'isola di Marettimo: Genova.

[20] Francini E, Messeri A (1956). L’isola di Marettimo nell’arcipelago delle Egadi e la sua vegetazione. Webbia.

[21] Monticelli T (1840). Del trattamento delle api in Favignana.

[22] Amico Statella VM. Lexicon Topographicum Siculum. In quo Siciliae Urbes, Oppida, cum diruta, tum extantia, Montes, Flumina, Portus, adjacentes Insulae, ac singula loca describuntur, illustrantur. Lexicon Topographicum Siculum. In quo Siciliae Urbes, Oppida, cum diruta, tum extantia, Montes, Flumina, Portus, adjacentes Insulae, ac singula loca describuntur, illustrantur’. P. Bentivenga, Panormi, vol. I, 374 pp. (1757); J. Pulejum, Catanae, vol. II, 384 pp. (1759); vol. III, 305 pp. (1760). Translated from Latin and annotated by G. di Marzo. In.; 1757-1760.

[23] Statistiche Demografiche ISTAT (2017 Data). http://demo.istat.it/pop2017/index3.html.

[24] Abate B, Incandela A, Nigro F, Renda P (1998). Plio-Pleistocene strike-slip tectonics in the Trapani Mts (NW Sicily). Boll Soc Geol It.

[25] Abate B, Lo Cicero G, Renda P (1992). Facies Carbonatiche ed evaporitiche del Trias superiore di Marettimo. Rend Soc Geol Ital It.

[26] Abate B, Ferruzza G, Incandela A, Renda P (1995). Tettonica trascorrente nelle Isole Egadi (Sicilia occidentale). Studi Geologici.

[27] Agnesi V, Macaluso T, Orrù P, Ulzega A (1993). Paleogeografia dell’Arcipelago delle Egadi (Sicilia) nel Pleistocene sup.-Olocene. Naturalista Sicil.

[28] Rivas-Martinez S (1995). Bases para una nueva classificacion bioclimatica de la Tierra. Folia Bot Matrit.

[29] Gianguzzi L, Scuderi L, Pasta S (2006). La flora vascolare dell’Isola di Marettimo (Arcipelago delle Egadi, Canale di Sicilia): aggiornamento ed analisi fitogeografica. Webbia.

[30] International Society of Ethnobiology code of ethics (with 2008 additions) [http://ise.arts.ubc.ca/global_coalition/ethics.php]

[31] Southeast Regional Network of Expertise and Collections [http://sernecportal.org/portal/s]

[32] Bartolucci F, Peruzzi L, Galasso G, Albano A, Alessandrini A, Ardenghi NMG, Astuti G, Bacchetta G, Ballelli S, Banfi E, Barberis G, Bernardo L, Bouvet D, Bovio M, Cecchi L, di Pietro R, Domina G, Fascetti S, Fenu G, Festi F, Foggi B, Gallo L, Gottschlich G, Gubellini L, Iamonico D, Iberite M, Jiménez-Mejías P, Lattanzi E, Marchetti D, Martinetto E, Masin RR, Medagli P, Passalacqua NG, Peccenini S, Pennesi R, Pierini B, Poldini L, Prosser F, Raimondo FM, Roma-Marzio F, Rosati L, Santangelo A, Scoppola A, Scortegagna S, Selvaggi A, Selvi F, Soldano A, Stinca A, Wagensommer RP, Wilhalm T, Conti F (2018). An updated checklist of the vascular flora native to Italy. Plant Biosystems - An International Journal Dealing with all Aspects of Plant Biology.

[33] Galasso G, Conti F, Peruzzi L, Ardenghi NMG, Banfi E, Celesti-Grapow L, Albano A, Alessandrini A, Bacchetta G, Ballelli S, Bandini Mazzanti M, Barberis G, Bernardo L, Blasi C, Bouvet D, Bovio M, Cecchi L, del Guacchio E, Domina G, Fascetti S, Gallo L, Gubellini L, Guiggi A, Iamonico D, Iberite M, Jiménez-Mejías P, Lattanzi E, Marchetti D, Martinetto E, Masin RR, Medagli P, Passalacqua NG, Peccenini S, Pennesi R, Pierini B, Podda L, Poldini L, Prosser F, Raimondo FM, Roma-Marzio F, Rosati L, Santangelo A, Scoppola A, Scortegagna S, Selvaggi A, Selvi F, Soldano A, Stinca A, Wagensommer RP, Wilhalm T, Bartolucci F (2018). An updated checklist of the vascular flora alien to Italy. Plant Biosystems - An International Journal Dealing with all Aspects of Plant Biology.

[34] MycoBank Database: Fungal databases, nomenclature and species banks

[35] Trotter RT, Logan MH, Etkin N (1986). Informant consensus: a new approach for identifying potentially effective medicinal plants. Plants in Indigenous Medicine & Diet.

[36] Heinrich M, Ankli A, Frei B, Weimann C, Sticher O (1998). Medicinal plants in Mexico: healers' consensus and cultural importance. Social Science & Medicine.

[37] Friedman J, Yaniv Z, Dafni A, Palewitch D (1986). A preliminary classification of the healing potential of medicinal plants, based on a rational analysis of an ethnopharmacological field survey among Bedouins in the Negev Desert. Israel. Journal of Ethnopharmacology.

[38] de Albuquerque UP, de Medeiros PM, de Almeida ALS (2007). Monteiro JM, de Freitas Lins Neto EM, de Melo JG, dos Santos JP: Medicinal plants of the caatinga (semi-arid) vegetation of NE Brazil: a quantitative approach. Journal of Ethnopharmacology.

[39] Di Martino A, Trapani S (1968). Flora e vegetazione delle isole di Favignana e Levanzo nell'Arcipelago delle Egadi. II. Levanzo. Lav Ist Bot e Giard Colon Palermo.

[40] Romano S, Tobia G, Gianguzzi L (2006). Rassegna della flora vascolare dell’Isola di Levanzo (Arcipelago delle Egadi, Canale di Sicilia). Informatore Botanico Italiano.

[41] Di Martino A, Trapani S (1967). Flora e vegetazione delle isole di Favignana e Levanzo nell'Arcipelago delle Egadi. II. Favignana. Lav Ist Bot e Giard Colon Palermo.

[42] Arcidiacono S, Napoli M, Pavone P (2003). Piante selvatiche d'uso popolare nel territorio di Bronte (Catania). Quad Bot Ambientale Appl.

[43] Aleo M, Cambria S, Bazan G (2013). Tradizioni etnofarmacobotaniche in alcune comunità rurali dei Monti di Trapani (Sicilia occidentale). Quad Bot Ambientale Appl.

[44] Guarrera PM (2009). Le piante nelle tradizioni popolari della Sicilia. In: Erboristeria Domani Gennaio.

[45] Barbagallo C, Grillo M, Meli R (1979). Nota sulle piante officinali spontanee e coltivate del territorio di Cesarò (Messina). Fitoterapia.

[46] Arcidiacono S, Napoli M (1999). P: Piante selvatiche utilizzate nella medicina e nella veterinaria popolari nel territorio di Bronte (Catania). Quad Bot Ambientale Appl.

[47] Pignatti S, Guarino R, La Rosa M: Flora d’Italia, vol. 1-4, 2nd edn. Bologna: Edagricole; 2017-2019.

[48] Calcara P (1851). Florula medica siciliana o esposizione delle piante indigene medicinali. Opera che fa seguito alla Farmacopea del Campana, con aggiunta di Michelotti.

[49] Arcidiacono S, Costa R, Marletta G, Pavone P, Napoli M (2010). Usi popolari dellepiante selvatiche nel territorio di Villarosa (EN–Sicilia Centrale). Quad Bot Ambientale Appl.

[50] Napoli M (2008). The plants, rituals and spells that 'cured' helminthiasis in Sicily. J Ethnobiol Ethnomed.

[51] Aleo M, Azzaro D, Cambria S, Bazan G (2020). Indagini etnobotaniche nel territorio di Paceco (Sicilia occidentale) Naturalista Sicil.

[52] Aleo N, Amato F, Aleo M (2011). Le piante tossiche della flora trapanese (Sicilia). Quad Bot Ambientale Appl.

[53] Ilardi V, Raimondo F (1992). L'uso tradizionale delle piante nella comunità rurale di Mezzojuso (Palermo). Quad Bot Ambientale Appl.

[54] Barbagallo C, Meli R, Savoca F, Nicotra M (2004). Indagine sugli usi popolari delle piante medicinali della Sicilia centro-orientale. Boll Accad Gioenia Sci Nat Catania.

[55] Maravigna C. Saggio di una flora medica catanese ossia catalogo delle principali piante medicinali che spontaneamente crescono in Catania e nei suoi contorni con la indicazione delle loro mediche azioni. Atti Accad Gioenia Sci Nat, Catania. 1827;2:67–120.

[56] Lentini F, Aleo M, Amenta R (1997). L’uso popolare delle piante nelle isole Egadi (Sicilia). Acta Phytotherapeutica.

[57] Bonomo R, Trapani S: Piante officinali nelle Egadi. Lavori dell’Istituto Botanico e del Giardino Coloniale di Palermo. In., vol. 25. Biblioteca delle Scienze Agrarie, Palermo, Italy; 1974: 195-233.

[58] Bertolino F. Indagine sugli usi tradizionali delle piante dell’Isola di Favignana (Egadi): Università degli Studi di Palermo; 1988.

[59] Rizza U. I Semplici di Favignana. Egadi e Natura, Litotipografia Abate, Paceco. Italy; 2001.

[60] Savo V, La Rocca A, Caneva G, Rapallo F, Cornara L (2013). Plants used in artisanal fisheries on the Western Mediterranean coasts of Italy. Journal of Ethnobiology and Ethnomedicine.

[61] Surico G (2020). Lampedusa: dall’agricoltura, alla pesca, al turismo. In.

[62] Cornara L, Smeriglio A, Frigerio J, Labra M, Di Gristina E, Denaro M, Mora E, Trombetta D (2018). The problem of misidentification between edible and poisonous wild plants: reports from the Mediterranean area. Food Chem Toxicol.

[63] Guarrera PM (2006). Usi e Tradizioni della Flora Italiana. Medicina Popolare ed Etnobotanica. Rome, Italy.: Aracne.

[64] Maestrini M, Molento MB, Mancini S, FSRd C, Furnari G, Serio D, Cornara L, Perrucci S (2021). Evaluation of the anthelmintic properties of a traditional remedy based on a mixture of red algae using an in vitro assay on gastrointestinal nematodes of donkeys. Open J Chemistry.

[65] Hooper D (1937). Useful plants and drugs of Iran and Iraq.

[66] Lansky EP, Paavilainen HM, Lansky S. Caper: the genus Capparis. In: Traditional Herbal Medicines for Modern Times. vol. 12: CRC Press; 2013. p. 52.

[67] Sala S, Pasta S, Maggiore V, La Mantia T (2020). Traditional use of wood in Sicily. In: Life on islands Biodiversity in Sicily and surrounding islands. Edited by La Mantia T, Badalamenti E, Carapezza A, Lo Cascio P, Troia A.

[68] Vaccaro V (2016). Nuova guida per Viaggiatori e Curiosi. Storia, Natura e Itinerari dell’Isola più lontana dell’arcipelago delle Egadi. Allegato a Il Giornale delle Egadi.

[69] La Mantia T, Carimi F, Di Lorenzo R, Pasta S. The agricultural heritage of Lampedusa (Pelagie Archipelago, South Italy) and its key role for cultivar and wildlife conservation. Ital J Agronomy. 2011;6(2):17.

[70] Tuttolomondo T, Licata M, Leto C, Savo V, Bonsangue G, Letizia Gargano M, Venturella G, La Bella S (2014). Ethnobotanical investigation on wild medicinal plants in the Monti Sicani Regional Park (Sicily, Italy). Journal of Ethnopharmacology.

[71] Geraci A, Amato F, Di Noto G, Bazan G, Schicchi R (2018). The wild taxa utilized as vegetables in Sicily (Italy): a traditional component of the Mediterranean diet. Journal of Ethnobiology and Ethnomedicine.

[72] Pasta S, La Rosa A, Garfì G, Marcenò C, Gristina AS, Carimi F, Guarino R (2020). An updated checklist of the Sicilian native edible plants: preserving the traditional ecological knowledge of century-old agro-pastoral landscapes. Front Plant Sci.

[73] Licata M, Tuttolomondo T, Leto C, Virga G, Bonsangue G, Cammalleri I, Gennaro MC, La Bella S (2016). A survey of wild plant species for food use in Sicily (Italy) - results of a 3-year study in four Regional Parks. Journal of Ethnobiology and Ethnomedicine.

[74] Gómez-Baggethun E, Reyes-García V, Olsson P, Montes C (2012). Traditional ecological knowledge and community resilience to environmental extremes: a case study in Doñana. SW Spain. Global Environmental Change.

[75] Barthel S, Crumley CL, Svedin U (2013). Biocultural refugia: combating the erosion of diversity in landscapes of food production. Ecology and Society.

