# Supplementary material for: Ethnobotany of the Aegadian Islands: safeguarding biocultural refugia in the Mediterranean
Source: J Ethnobiol Ethnomed. 2021 Jul 28;17:47. doi: 10.1186/s13002-021-00470-z (PMC8320050; doi:10.1186/s13002-021-00470-z)
Supplement: Supplementary file 1 — Additional file 1. Ethnobiological uses of local taxa. Data table reporting the specific uses of local taxa documented in the present work and comparison to prior publications in the region. (.pdf file format). [file 13002_2021_470_MOESM1_ESM.pdf]

Additional File 1. Ethnobiological uses of local taxa. Taxa and specific uses that were also cited in prior literature are denoted in with reference the source. Documented use citations for practices no longer in use today are denoted with “[text]” to signify past use. Voucher codes are provided for wild species (cultivars excluded).

Abbreviations: **St:** Status. C: cultivated; SC: semi-cultivated; W: wild; P: purchased. **Local Name:** Local names reported by Aegadian islanders in local dialect are reported here. “—“ indicates that no local name was cited. **English Common Name:** “—“ indicates that no English common name is available. Note: English common names were not cited by informants; these are included in the table as a general reference. **UV_c:** Use-value Citation Index. This index measures the relative importance of each species based on its reported use by informants. **PU:** Parts Used. AP: Aerial parts. Ba: Bark. Br: Branches. Bu: Bulbs. Co: Cones. Fbr: Flowering branch. Fl: Flowers. Fr: Fruits. Fro: Fronds. Inf: Inflorescence. L: leaves. La: latex. Lg: leaf gel. No: node. Oil: Oil. Pa: Paddle. Res: Resin. Rh: Rhizomes. Ro: Roots. Se: Seeds. Sh: Shoots. Sp: Sporophore. St: Stem. Sti: stigma. Tu: Tubers. Wo: wood. WP: whole plant. YL: young leaves. Ysb: Young stems and branches. **Local preparation and use:** Those uses that were also cited in prior reports on Aegadian ethnobotany are denoted by reference source. **U_{is}:** Total number of use citation reports by individual informants. **FL:** Fidelity Level %. This measure is useful for highlighting the central role of each taxa. Any taxa with ≤3 total use citations is excluded from this analysis due to limitations of this tool, and is denoted with —. **PC:** Prior Citation. Other reports of similar ethnobotanical applications of the plant or fungal resource. **Different Uses Reported:** Other reports previously referred for the same species in the ethnobotanical literature of the Aegadian Islands.

Taxa & Voucher Code	St	Local Dialect Name	English Common Name	UV_c	PU	Local preparation and use	U_{is}	FL	PC	Different Uses Reported in PC
**FUNGI**										
**Boletaceae**										
Leccinellum corsicum (Rolland) Bresinsky & Manfr. Binder	W	funcia di rusedda	—	0.02	Sp	Food: boiled and eaten. Harvested from autumn to winter in scrubland with *Cistus* species.	1	—	—	—
**Pleurotaceae**										
Pleurotus eryngii (DC.) Quél. var. *eryngii*	W	funcia i pani cauru	king oyster mushroom	0.04	Sp	Food: Cooked and eaten. Harvested from autumn to winter in fields.	2	—	—	—
Pleurotus eryngii (DC.) Quél. var. *ferulae* (Lanzi) Sacc. CQ-878	W	funcia i fella	king oyster mushroom	0.29	Sp	Food: Cooked (boiled, grilled or roasted) and eaten. Harvested from autumn to winter, grows at the base of dried remnants of *Ferula communis* plants. Preferred edible fungus/considered the most delicious of the islands.	14	100%	—	—
Pleurotus opuntiae (Durieu & Lév.) Sacc.	W	funcia i fucurinia	oyster mushroom	0.02	Sp	Food: Cooked and eaten. Found growing near *Opuntia ficus-indica* in autumn to winter.	1	—	—	—
**Suillaceae**										
Suillus collinitus (Fr.) Kuntze CQ-856	W	pinarolu; funci i pinu	—	0.06	Sp	Food: Cooked and eaten. Found in autumn to winter growing in the pine forests.	3	—	—	—
**PLANTAE**										
**Acanthaceae**										
Acanthus mollis L. CQ-609	W	vrancaursina;	bear's breeches; sea dock	0.08	L	Ethnoveterinary: Feed for livestock	4	100%	[1, 2]	General Health: L and Ro [3, 4]
**Amaryllidaceae**										
Allium commutatum Guss.	W	agghia sarbaggia	wild onion	0.10	Bu	Food: Harvested before flowering, eaten as a condiment/flavoring	5	100%	—	—

Taxa & Voucher Code	St	Local Dialect Name	English Common Name	UV_c	PU	Local preparation and use	U_{is}	FL	PC	Different Uses Reported in PC
Allium sativum L.	C	agghia	garlic	0.29	Bu	Dermatological: Mix with hot baked bread and bay leaves, apply topically to treat skin infections (cysts, furuncles, boils)	4	29%	—	—
						Dermatological: Lenitive, moisturizing effects on the skin when treating damaged skin, burns, cold sores or cracked lips	5	36%	—	—
						Gastrointestinal: Rub the bulb onto the belly button as a vermifuge against intestinal worms; or smash and place in cloth to leave on abdomen overnight	3	21%	—	—
						Gastrointestinal: String the bulbs together as a necklace to be worn by children as a vermifuge against intestinal worms	2	14%	—	—
**Anacardiaceae**										
Pistacia lentiscus L. CQ-584; CQ-920	W	stincu; listincu	mastic tree	0.40	Ysb	Household: Basket making; creation of border for mule saddles	1	5%	—	Dermatological: cosmetic, Res [3, 4]
					St	Household: The woody stem was used as a central pivot in the construction of "furrizi" – a traditional stool made of dried *Ferula communis* stems	1	5%	—	—
					Wo; Br	Household: Firewood to heat ovens for bread baking. Also sold from one island to another, or to the mainland (Trapani).	17	89%	—	—

Taxa & Voucher Code	St	Local Dialect Name	English Common Name	UV_c	PU	Local preparation and use	U_{is}	FL	PC	Different Uses Reported in PC
Rhus coriaria L. CQ-603	W	summaccu	Sicilian sumac	0.15	L	Oral health: Decoction for gingival inflammation	1	14%	[3]	Dermatological: hemostatic; General Health: fever, Ba, L, Fr [3]
					L	Maritime: Dried and used to dye fishing nets	1	14%	—	—
					Br	Household: Firewood to heat ovens for bread baking	1	14%	—	—
					L; Ba	Household: Dried and sold as a dye	4	57%	—	—
**Apiaceae**										
Crithmum maritimum L. CQ-597; CQ-902	W	finucchieddu di mari; finocchiu marinu	samphire; rock samphire; sea fennel	0.02	L	Dermatological: Decoction made and cloth soaked in resulting liquid, topically applied to furuncles	1	—	—	Gastrointestinal: vermifuge, eupeptic, digestive; Urological: diuretic, Fr, Ro [2-4]
Daucus carota L. subsp. *carota* CQ-911	W	vastunaca; bastunaca	wild carrot	0.08	Ro	General health: medicinal food, also diuretic; sold [in past]	4	100%	[3, 4]	Gastrointestinal: vermifuge; Women's health: galactagogue; Fr, Ro [3, 4]
Eryngium campestre L.	W	spini viola; pani cavuru	field eryngo	0.02	WP	Household: Indicator species, where edible fungi are found (similar to *fungi ferla*, but broader)	1	—	—	Urological: diuretic, Ro [3, 4]
Ferula communis L. subsp. *communis* CQ-589	W	fella; ferla manza; ferla	giant fennel	0.48	St	Household: Construction of small stools and tables	9	39%	—	—
						Household: As a handle for knitting needles	4	17%	[1, 2]	—
						Household: For sharpening barber blades	4	17%	—	—

Taxa & Voucher Code	St	Local Dialect Name	English Common Name	UV_c	PU	Local preparation and use	U_{is}	FL	PC	Different Uses Reported in PC
					AP	Household: Dried aerial parts burned as firewood	6	26%	—	—
Foeniculum vulgare Mill. subsp. *vulgare* CQ-599	W	finucchieddu sarbaggiu	sweet fennel	0.79	Se	Food: Seasoning	6	16%	[1, 2]	—
						Food: Seasoning for cured sausage	4	11%	[1, 2]	—
						Gastrointestinal: Eaten as a carminative and for stomachache or heartburn	5	13%	[2-4]	—
					AP	Food: Used to make a local specialty dish *polpette di finochietto*, cooked, cut up and mixed with bread crumbs, salt and pecorino or ricotta	5	13%	[1]	—
					AP; Se	Food: Used to make a local specialty dish *pasta con le sarde*	3	8%	—	—
					L; AP, Fr	General health: Boil to prepare a tisane to drink as a depurative	1	3%	[2-4]	—
					St	Food: Tip of fresh stem cut and added to brine lacto-fermentation of olives as a flavoring agent	1	3%	[1, 2]	—
					L; AP; Fr	Food: Boiled and eaten	12	32%	[1, 2]	—
					AP	Food: Eaten fresh	1	3%	—	—
Smyrnium olusatrum L. CQ-607	W	lisciannaru	alisanders; horse parslet	0.04	—	Household: Used to dye fishing nets brown [past use]	1	—	—	—
					Wo	Household: Firewood to heat ovens for bread baking	1	—	—	—
Thapsia garganica L. subsp. *garganica* CQ-619	W	fella ianca; firlazzu	drias plant	0.08	St	Maritime: Use scape of stems to get octopus out of its den	4	100%	—	Nuisance: revulsive, vesicant, Ro [3]

Taxa & Voucher Code	St	Local Dialect Name	English Common Name	UV_c	PU	Local preparation and use	U_{is}	FL	PC	Different Uses Reported in PC
**Apocynaceae**										
Nerium oleander L. subsp. *oleander*	C	—	oleander	0.02	Fl	Household: Fresh flowers placed on windowsills to deter insects, such as mosquitos, from entering the home	1	—	[1, 3]	Respiratory: resolve sneezing, L, Ba [3]
Periploca angustifolia Labill. [= *Periploca laevigata* Aiton *subsp. angustifolia* (Labill.) Markgr.] CQ-602	W	scornabeccu	—	0.15	Br; Wo	Household: Firewood, also used as firewood to heat ovens for baking bread	6	86%	—	—
					Fr; Se	Dermatological: The inner white fuzzy/feathery seeds are applied to lacerations as a hemostatic	1	14%	—	—
**Araceae**										
Arisarum vulgare Targ. Tozz. subsp. *vulgare* CQ-915	W	aricchia ri cani; anzaru	friar's cowl; larus	0.25	Rh	Ethnoveterinary: Fodder for pigs; encourage weight gain in pigs (makes better fatty meat)	11	92%	—	—
					Fl	Household: Environmental indicator – when in flower, it is the best time to collect wild edible fungi	1	8%	—	—
**Arecaceae**										
Chamaerops humilis L.	W	giummara	European fan palm	0.33	L	Household: Dried leaves used to make small brooms for cleaning ovens and for cleaning the pots used to make ricotta	9	56%	[2]	—
						Household: Woven to create *coffe* (artisanal baskets)	5	31%	[2]	—
						Household: Woven to create bags	1	6%	—	—
						Household: Used to create *muscaloru*, a device for blowing air onto the fire	1	6%	—	—

Taxa & Voucher Code	St	Local Dialect Name	English Common Name	UV_c	PU	Local preparation and use	U_{is}	FL	PC	Different Uses Reported in PC
**Asparagaceae**										
Agave americana L. subsp. *americana* CQ-600	W	zabbara	century plant, sentry plant, American aloe	0.90	Inf	Household: Used as general firewood for heating ovens to bake bread	2	5%	—	—
						Household: Used as a support post to build a shade structure called *friscale*	1	2%	—	—
					Ro	Household: Used to build stools or seats	1	2%	—	—
					L	Household: Leaves were cut into four parts and left to dry, and the resulting fiber was used as cordage in agricultural activities	7	16%	[1, 2]	—
						Maritime: Leaves were cut into four parts and left to dry, and the resulting fiber was used as cordage in fishing activities	3	7%	—	—
						Household: Leaves were cut into four parts and left to dry, and the resulting fiber was used to tie bundles of hay or to stuff chairs	12	28%	—	—
						Household: The apical spine of the leaf is used as fiber or cordage	1	2%	—	—
						Household: The apical spine of the leaf is used as a needle for sewing or for eating land snails	3	7%	—	—
						Household: Ashes made from burning the dry leaves are used to wash white clothes as a whitening agent	5	12%	[1]	—

Taxa & Voucher Code	St	Local Dialect Name	English Common Name	UV_c	PU	Local preparation and use	U_{is}	FL	PC	Different Uses Reported in PC
						Dermatological: Fresh leaf is cut in half and the inner gel side applied to soothe burn wounds	1	2%	—	Urological: depurative, diuretic, L, Ro [3, 4]
						Musculoskeletal/Neurological: The outer leaf epidermidis is removed, and the inner leaf epidermidis is rubbed on the affected area, usually the shoulders, to alleviate back pain. The process stings the skin. Plants growing closest to the sea are most effective.	2	5%	—	—
					WP	Household: Planted in a row to divide property	5	12%	[1]	—
Agave sisalana Perrine ex Engelm.	W	zabbara	sisal hemp	0.21	Inf	Household: Used as firewood for heating ovens to bake bread	2	20%	—	—
						Household: Used as a support post to build a shade structure called *friscale*	1	10%	—	—
					Ro	Household: Used to build stools or seats	1	10%	—	—
					L	Household: Leaves were cut into four parts and left to dry, and the resulting fiber was used as cordage in agricultural activities	1	10%	—	—
						Household: Leaves were cut into four parts and left to dry, and the resulting fiber was used to tie bundles of hay or to stuff chairs. The cordage of this species was considered superior to that of *A. americana*.	2	20%	—	—

Taxa & Voucher Code	St	Local Dialect Name	English Common Name	UV_c	PU	Local preparation and use	U_{is}	FL	PC	Different Uses Reported in PC
						Maritime: Leaves were cut into four parts and left to dry, and the resulting fiber was used as cordage in fishing activities. This species was considered superior to that of *A. americana* for this purpose because of the lack of spines along the leaf margin.	3	30%	—	—
Asparagus acutifolius L. CQ-593	W	sparaci	wild asparagus	0.40	Sh	Food: Boiled and eaten; seasoned with olive oil and lemon juice, or subsequently fried	15	79%	[1, 2]	—
						Urological: Decoction drunk to treat kidney stones	2	11%	[1-4]	—
						Urological: Decoction made, bread dipped into the decoction and eaten as a diuretic	2	11%	—	—
Charybdis pancration (Steinh.) Speta [= *Drimia pancration* (Steinh.) J. C. Manning & Goldblatt.] CQ-595	W	cipuddazzu	red squill; sea squill; sea onion	0.04	Bu	Dermatological: The fresh bulb is crushed and mixed with bicarbonate, and then topically applied as a hemostatic for wounds	2	—	—	—
**Asphodelaceae**										
Aloe spp.	C	—	aloe	0.06	Lg	Dermatological: Leaves are sliced to expose the inner gel and topically applied to treat burn wounds	3	—	—	—
Asphodelus ramosus L.	W	vastuni i San Giuseppi ; purrazzu	branched asphodel	0.13	Infl	Household: Ritual use as floral decoration of the altar during the religious festival of San Giusseppe	3	50%	—	—
					St	Household: Firewood	2	33%	—	—

Taxa & Voucher Code	St	Local Dialect Name	English Common Name	UV_c	PU	Local preparation and use	U_{is}	FL	PC	Different Uses Reported in PC
					Ro	Ethnoveterinary: Fodder for rabbits	1	17%	—	Dermatological: cosmetic, Ro [4]
**Asteraceae**										
Anthemis secundiramea Biv. [incl. subsp. *intermedia* (Guss.) R. Fern.]	W	—	prostrate chamomile	0.10	Inf	General health: Infloresences are sun-dried and stored for later use; made into tisane/infusion as relaxant/sedative	3	60%	—	—
						General health: Infloresences are sun-dried and stored for later use; made into tisane/infusion as anti-inflammatory	2	40%	—	—
Artemisia arborescens (Vaill.) L. CQ-598; CQ-867	W	erva janca; erba vagnu; vagnu	tree wormwood	0.46	AP	Dermatological: Ritual washing and purification of the hands as a perfume for the festival of San Giuseppe. The perfumed water is made from a cold water maceration of the aerial parts overnight. Rose petals and mint leaves are also added.	3	14%	—	Gastrointestinal: digestive, vermifuge, cholagogue, tonic, bitter, L [3]
						Dermatological: A cold water infusion of the aerial parts is used in ritual bathing of an infant or newborn, especially for the first bathing of the newborn. This can be repeated up to three times (3 baths) maximum. It is also considered an antiseptic and cosmetic perfume for the infant's skin. If the infant is a girl, the bath water is tossed into the bucket used for	15	68%	[1]	—

Taxa & Voucher Code	St	Local Dialect Name	English Common Name	UV_c	PU	Local preparation and use	U_{is}	FL	PC	Different Uses Reported in PC
						washing clothes when done in order to bring prosperity to her life in the home and housework. If a boy, the used bath water is tossed in the street as a symbol of the boy's future path to go out into the world and find a job. Some also note that the first person to pass by after the water is tossed (rich or poor person) will determine the future success of the boy.				
						Dermatological: Infusion or decoction used in refreshing baths for adults	2	9%	—	—
					Wo	Household: Woody parts used as firewood	1	5%	—	—
					Le	Household: The aromatic leaves were placed in the coffin of the deceased as a perfume	1	5%	—	—
Centaurea calcitrapa L. CQ-629	W	spinaprocchia	red star thistle; purple starthistle	0.04	Inf	Food: Base of inflorescence boiled and eaten (famine food)	2	—	—	General health: fever; Gastrointestinal: eupeptic; Urological: diuretic, L, Inf, Ro, Ro [3, 4]
Crepis vesicaria L. subsp. *vesicaria*	W	—	beaked hawk's beard	0.52	Le	Food: Boiled and eaten as a side dish; may be left to soak in water before eating to remove some of the bitter flavor	18	72%	[1]	—
						General health: The bitter water remaining after boiling	3	12%	—	—

Taxa & Voucher Code	St	Local Dialect Name	English Common Name	UV_c	PU	Local preparation and use	U_{is}	FL	PC	Different Uses Reported in PC
						the leaves to eat is drunk for general health promotion as a general depurative. Follows the proverb to keep bitter things precious: "*Cosi amari, tenili cari*" or "*tieni caro tutto cio che ha un sapore amaro*".				
						General health: The bitter water remaining after boiling the leaves to eat is drunk to treat stomachaches	1	4%	—	—
						Cardiovascular: The bitter water remaining after boiling the leaves to eat is drunk to treat anemia	1	4%	—	—
						Food: Used as a substitute for other cicoria (*Cichorium intybus*) as the other species is rarely found locally (on Levanzo).	1	4%	—	—
						Food: Boiled and dressed with olive oil and garlic. Not recommended for breastfeeding mothers as it is believed to make the milk very bitter for the infant.	1	4%	—	—
Dittrichia viscosa (L.) Greuter CQ-617	W	panicaseddu; erva di San Giuvanni	false yellowhead; sticky fleabane	0.02	Inf	Dermatological: Flowers are dried and ground into a powder; this is topically applied as a hemostatic	1	—	—	Dermatological: astringent, vulnerary; Gastrointestinal: antidiarrheal, Fl [3]
Galactites tomentosus Moench CQ-564	W	spina ianca	Mediterranean thistle; boar thistle	0.02	St	Food: The soft stems are boiled and eaten	1	—	—	—

Taxa & Voucher Code	St	Local Dialect Name	English Common Name	UV_c	PU	Local preparation and use	U_{is}	FL	PC	Different Uses Reported in PC
Glebionis coronaria (L.) Cass. ex Spach CQ-905	W	maiu	garland chrysanthemum; crown daisy	0.04	Inf	[Household: In spring, the flowers were strewn in the home (under the bed, in drawers) as a celebration that spring has come.]	1	—	—	—
						Household: As a childhood game, the pollen would be rubbed on the face to mimic the yellow tint of the dead	1	—	—	—
Hyoseris radiata L.	W	cardedda i serpi	perennial hyoseris	0.13	Le	Urological: Decoction of leaves drunk to treat kidney stones	5	83%	[1, 2]	Dermatological: topical for skin disease; Food, Ro, WP [1, 2]
						Urological: Drunk to treat prostate problems	1	17%	—	—
Jacobaea maritima (L.) Pelser & Meijden subsp. *sicula* N.G.Passal., Peruzzi & Pellegrino CQ-592	W	sapunara	silver ragwort	0.10		Maritime: The fresh parts of the plant were put on a blanket used to cover boats so that their wood could keep cool throughout the summer and not get damaged.	1	20%	—	—
					Wo	Household: Used in household furniture construction, especially for beds	2	40%	—	—
						Household: Firewood	2	40%	—	—
Notobasis syriaca (L.) Cass.	W	spini viola	Syrian thistle	0.04	AP	Food: Eaten raw or boiled	2	—	—	Urological: diaphoretic, diuretic, tonic, Rh [4]
Reichardia picroides (L.) Roth	W	caccialebbra	common brighteyes	0.02	Le	Food: Boiled and eaten	1	—	—	—
Scolymus grandiflorus Desf.	W	spini cardiddi; scoddri; spina di cardiddi	golden thistle	0.27	Inf	Food: Flowers eaten raw as a game	5	62%	[2]	—

Taxa & Voucher Code	St	Local Dialect Name	English Common Name	UV_c	PU	Local preparation and use	U_{is}	FL	PC	Different Uses Reported in PC
					St	Food: Soft stem boiled and seasoned with olive oil and lemon juice and eaten	8	38%	—	—
Silybum marianum (L.) Gaertn. CQ-580	W	spina n'incannausa	milk thistle; blessed milk thistle	0.13	Se	General health: Seeds are crushed in milk or water and left to soak overnight; the macerate is drunk to promote general health	4	67%	—	General health: fever, tonic; Urological: diuretic, WP [3]; Cardiovascular: hypotensive; Gastrointestinal: eupeptic for liver disease, Ro [4]
					St	Food: The soft stem at the base of the inflorescence is cleaned and cooked, then eaten like an artichoke	2	33%	—	—
Sonchus oleraceus (L.) L. CQ-633	W	cardedda manza; cardedda; cardedda di serpi	common sowthistle	0.83	Le	Food: Tender young leaves eaten raw in salads	16	40%	—	—
						Food: Older leaves (those considered not young and tender enough for raw consumption) are boiled and eaten	23	58%	—	—
						Urological: Made into a decoction, drunk once cooled to treat kidney stones	1	3%	[3, 4]	—
**Boraginaceae**										
Borago officinalis L. CQ-553	W	burrania; burragu; vurrania	common borage	0.71	Le	General health: Leaves boiled and eaten as a depurative health food. It can be eaten alone or as a condiment to rice or pasta. Sometimes used as	20	59%	[2, 3]	Dermatological: skin burns, Le [1, 2]; Dermatological: acne, Le [4]

Taxa & Voucher Code	St	Local Dialect Name	English Common Name	UV_c	PU	Local preparation and use	U_{is}	FL	PC	Different Uses Reported in PC
						ravioli filling. Perceived health benefits are more accentuated than that for *cicoria*.				
						General health: Leaves used to be collected on the first Friday of March, dried, and stored in a cloth for use for many purposes (panacea)	5	15%	—	—
						Ethnoveterinary: Decoction of dried leaves prepared with a root of *gramigna*, and olive oil is added at the end of the preparation. Given to cattle and sheep suffering from digestive ailments to drink.	3	9%	—	—
						Ethnoveterinary: Dried leaves used to make a decoction given to livestock (cattle, sheep, mules) to drink as a refreshing beverage	6	18%	—	—
Cynoglossum creticum Mill. CQ-868	W	lingua ri cani	blue hound's tongue	0.06	Le	Dermatological: Fresh leaves are mashed with salt and topically applied as a suppurative for abscesses	1	—	[2, 4]	Neurological: decoction as sedative, Ro [4]
						Dermatological: Fresh leaves are mashed with salt and topically applied to bruises	1	—	—	—
						Dermatological: Fresh leaves applied as a cataplasm to promote wound healing	1	—	—	—
**Brassicaceae**										
Brassica rapa L. subsp. *campestris* (L.) Clapham	W	qualeddru	turnip rape; field mustard	0.02	Le	Food: Boiled, and then mash and fry before eating	1	—	—	—

Taxa & Voucher Code	St	Local Dialect Name	English Common Name	UV_c	PU	Local preparation and use	U_{is}	FL	PC	Different Uses Reported in PC
Diplotaxis erucoides (L.) DC. subsp. *erucoides* CQ-632	W	sinacciola	white wall-rocket	0.08	Le	Food: When the sheep graze on this, their milk tastes similar to the plant	4	100%	—	—
Diplotaxis tenuifolia (L.) DC. CQ-630	W	rucola sarbaggia; brucculiddo	perennial wall-rocket	0.10	Le	Food: Eat with meat or as a side salad	5	100%	—	—
Hirschfeldia incana (L.) Lagr.-Fossat subsp. *incana*	W	razza; qualeddru	shortpod mustard; hoary mustard	0.15	Le; St	Food: Leaves and tender stems are boiled and eaten	7	100%	—	—
Matthiola incana (L.) W.T. Aiton subsp. *incana* CQ-903	W	barco; barcu	hoary stock	0.08	AP	Household: Flowering aerial parts are used as an altar decoration for the festival of San Giuseppe; also used to decorate the festival scepter	4	100%	—	—
Raphanus raphanistrum L. subsp. *raphanistrum*	W	razzi; razza; razza amara; ravanastru	wild radish	0.21	Le	Nuisance: Prevent livestock from eating it; when cattle eat it they have blood in their urine	5	50%	—	Musculoskeletal: anti-rheumatic, Se [3]
						Food: Basal leaves boiled and eaten	2	20%	[1, 2]	—
						Food: Young tender leaves eaten raw as a bitter salad ingredient	1	10%	—	—
						Ethnoveterinary: Fodder for chickens	1	10%	—	—
					St	Food: Tender stems are boiled and seasoned with olive oil and vinegar	1	10%	—	—
**Cactaceae**										
Opuntia ficus-indica (L.) Mill. CQ-625	W	ficurinia	barbary fig; prickly pear fig	0.85	Fl	Urological: 2-3 dry flowers are boiled in a small pot of water, and the decoction drunk as a diuretic; anti-inflammatory for the urinary tract	20	49%	[1, 2, 4]	—

Taxa & Voucher Code	St	Local Dialect Name	English Common Name	UV_c	PU	Local preparation and use	U_{is}	FL	PC	Different Uses Reported in PC
						Gastrointestinal: Decoction of dried flowers drunk to treat stomachache	2	5%	[2]	—
						Cardiovascular: Decoction of flowers drunk to treat hypertension	1	2%	[4]	—
					Fr	Food: Fruit is boiled to make marmalades; use the masculine fruit because it has less seeds to remove.	2	5%	[1, 2]	Dermatological: skin disease, Fr [4]
						Food: The first fruiting of the cactus were removed in a process called "scuzzulunare", in which the fruits were knocked off the cactus and fed to pigs. These fruits were called "bastarduna", and were not eaten by humans. The second fruiting is sweeter and eaten raw, made into marmalades, or sold – shipped off the island.	6	15%	—	—
					Pa	Gastrointestinal: The spines are removed from the paddle and it is then sliced in half to expose the inner gel. The paddle is then roasted over a fire until hot, and then placed onto the stomachache while warm to treat stomachache and abdominal heat using the heat. At one time, this was left to hang on the windowsill until it dried or "died", symbolic of the disease resolving.	6	15%	[4]	—

Taxa & Voucher Code	St	Local Dialect Name	English Common Name	UV_c	PU	Local preparation and use	U_{is}	FL	PC	Different Uses Reported in PC
						General health: The young paddle is cleaned and split in half, then placed gel-side to the breast of a nursing mother to help the weaning process (to dry up the milk)	1	2%	—	—
						Otolaryngological/Respiratory: A syrup is made of the inner paddle gel and drunk to treat cough	1	2%	[4]	—
						Ethnoveterinary: Feed young paddles to livestock	1	2%	—	—
					WP	Household: Cacti were planted in a row to serve as a wind barrier for gardens, but were also used for a number of other purposes described above	1	2%	—	—
**Capparaceae**										
Capparis orientalis Veill. [= *Capparis spinosa* L. subsp. *rupestris* (Sm.) Nyman] CQ-587	W	chiappara; chiappara sarvaggia	caper	0.42	Fl	Food: Unripe flower buds are conserved under salt and used in salads and other dishes as a seasoning	20	100%	[1-3]	Gastrointestinal**:** emetic; General Health: anti-malarial, Le [4]; Gastrointestinal: eupeptic, depurative, antiemetic, Ro; Gastrointestinal: tonic; Musculoskeletal: anti-arthritic, AP [3]
**Caprifoliaceae**										
Lonicera implexa Aiton subsp *implexa* CQ-650	W	u trono;	European honeysuckle; woodbine	0.10	AP	General health: Infusions drunk for general promotion of health and well-being	5	100%	[1, 2]	—

Taxa & Voucher Code	St	Local Dialect Name	English Common Name	UV_c	PU	Local preparation and use	U_{is}	FL	PC	Different Uses Reported in PC
**Chenopodiaceae**										
Beta vulgaris L. subsp. *maritima* (L.) Arcang. CQ-907	W	giri; gira marina; gira	sea beet	0.33	L	Food: Boiled and consumed as a green vegetable	16	100%	[1, 2]	General Health: L and Ro [1-3]
**Cistaceae**										
Cistus creticus L. subsp. *creticus* CQ-889; CQ-873	W	mpiccicalora; ruseddra	rock rose	0.19	Wo	Household: Firewood	8	89%	—	Nuisance: balsamic revulsive [3]
					St	Household: The woody stem was used as a central pivot in the construction of “furrizi” – a traditional stool made of dried *Ferula communis* stems.	1	11%	—	—
Cistus salviifolius L.	W	ruseddra	sage-leaved rock rose; Gallipoli rose	0.08	Wo	Household: Firewood	4	100%	—	—
Cistus monspeliensis L.	W	ruseddra	Montpellier cistus; rockrose	0.08	Wo	Household: Firewood	4	100%	—	—
**Convolvulaceae**										
Convolvulus arvensis L.	W	curriola	field bindweed	0.08	AP	Ethnoveterinary: Fodder for chickens	4	100%	[1, 2]	Gastrointestinal: purgative; Dermatological: skin disease, AP [4]
Convolvulus althaeoides L.	W	—	mallow bindweed	0.02	Fl	Food: Snack food during childhood; suck on back of flower for sweet nectar	1	—	—	Gastrointestinal: purgative; General Health: antipyretic, AP [3, 4]
**Cucurbitaceae**										
Ecballium elaterium (L.) A. Rich. CQ-899	W	citruleddu; muluni sarbaggiu	squirting cucumber	0.06	Fr	Nuisance: If the seeds shoot into the eyes, this may require a trip to the hospital	3	—	—	Gastrointestinal: purgative, Ro [3, 4]
**Ericaceae**										

Taxa & Voucher Code	St	Local Dialect Name	English Common Name	UV_c	PU	Local preparation and use	U_{is}	FL	PC	Different Uses Reported in PC
Arbutus unedo L.	C	—	strawberry tree	0.10	Fr	Food: eaten raw	5	100%	—	—
Erica multiflora L. subsp. *multiflora* CQ-594	W	alenciu;	Mediterranean heath	0.19	Wo	Household: Firewood	5	56%	—	Urological: diuretic, depurative; Neurological: sedative; Musculoskeletal: antirheumatic, Fl [3]
					Fl	Household: Optimal for production of local honey	4	44%	—	—
**Euphorbiaceae**										
Euphorbia dendroides L. CQ-596; CQ-918	W	camarruni; latticammaruni	tree spurge	0.58	St; La	Maritime: The stems are cut and the emerging latex was used to catch fish. This practice is known as "*attassare i mari*". The branches were placed in a hemp sack along with the leaves of *Ferula communis* and then beaten with a stick. This latex sack was then immersed in the sea. If stuck into an octopus den, the octopus emerges in escape from the irritant latex and is easily caught.	4	14%	—	—
					La	Maritime: Latex of the crushed plant is mixed with salted fish as bait. When fish approach the bait, they are "blinded" by the poison latex and easily caught.	2	7%	—	—
						Maritime: Latex of the crushed plant is used to stun fish. This practice is known as "*attassare*	3	11%	—	—

Taxa & Voucher Code	St	Local Dialect Name	English Common Name	UV_c	PU	Local preparation and use	U_{is}	FL	PC	Different Uses Reported in PC
						i mari". A system of nets is set up surrounding the area where the practice takes place in order to catch the stunned fish. Some noted that this practice has been replaced with the use of the agricultural chemical "verderame" – a copper pesticide – in its place.				
						Nuisance: To wean a baby from a nursing mother, a small amount of latex is placed on the mother's nipple to discourage the infant from nursing.	1	4%	—	—
						Nuisance: If the irritant latex gets into the eyes, it can be cleaned out with mother's milk. This helps lessen the burning pain. In cases of emergency, breast milk was also requested by non-family members to treat the affected eye.	3	11%	—	—
						Nuisance: Latex is an irritant to the skin and eyes, considered a poison	3	11%	—	Dermatological: treat warts, La [4]
					Br; St	Household: The dry branches and stems are used as firewood for the home. Once free of latex (dried) women could break it into small pieces for firewood.	12	43%	—	—
**Fabaceae**										

Taxa & Voucher Code	St	Local Dialect Name	English Common Name	UV_c	PU	Local preparation and use	U_{is}	FL	PC	Different Uses Reported in PC
Anagyris foetida L.	W	carrubbeddu anzale	stinking bean trefoil	0.08	WP	Nuisance: Poisonous	4	100%	—	Gastrointestinal: laxative, vermifuge, emmenagogue, emetic, AP [3]
Astragalus boeticus L.	W	cafè messicanu; cicirimignulo	Swedish coffee	0.17	AP	Ethnoveterinary: Fodder/forage for cattle; highly preferred wild food for cattle	4	50%	[1, 2]	—
					Se	Food: Eaten raw as a snack, or when playing games	4	50%	—	—
Ceratonia siliqua L. CQ-618; CQ-886	W	carrubbu	carob tree; locust bean	0.42	Fr	Otolaryngological/Respiratory: Prepared as a syrup with lemon leaves, dried figs and bay leaves. Drunk as an antitussive.	6	30%	—	Dermatological: emollient, Fr; astringent, Ba Le, Fr [3]; Respiratory: decoction for respiratory disease, Fr [1, 2, 4]; Respiratory: expectorant, Se [3]
						Otolaryngological/Respiratory: Boiled for a long time with dried fava beans, sugar and honey, with regular stirring. One small spoon taken in morning and night for children, or one large spoonful morning and night for adults as an antitussive.	1	5%	—	—
						Ethnoveterinary: Given to various livestock to eat (cattle, horses, sheep, donkeys) and also as a galactagogue.	3	15%	[1, 2]	—

Taxa & Voucher Code	St	Local Dialect Name	English Common Name	UV_c	PU	Local preparation and use	U_{is}	FL	PC	Different Uses Reported in PC
						Food: Eaten during wartime	2	10%	—	—
						Food: Eaten fresh [very common in past]	3	15%	—	—
						Food: Mixed with figs and used in making local sweets “cudduredde”	1	5%	—	—
						Food: Wine (*Vitis vinifera*) was diluted with water to make “peri”, and then mixed with a decoction of 4-5 carob fruits boiled in 4-5 liters of water. This is left to ferment and the volume reduces, yielding a final product of “carrubbata”	2	10%	—	—
						Food: Roasted in oven and eaten	1	5%	—	—
						Household: Fruit pods and seeds are mixed with hay in the livestock stall to produce a nice smell	1	5%	—	—
Lotus edulis L.	W	curnicchia; cornicchio; curnicchiu	edible birdsfoot trefoil	0.19	Fr	Ethnoveterinary: Forage for livestock	1	11%	—	—
					Se	Food: Snack food, like a candy, for children	8	89%	—	—
Lathyrus oleraceus Lam. subsp. *biflorus* (Raf.) H.Schaef., Coulot & Rabaute [=*Pisum sativum* subsp. *elatius* Asch. & Graebn.]	W	chiffella ; pisedda sarbaggia	garden pea	0.10	Se	Food: Cooked in egg frittatas	5	100%	—	—
Vicia faba L.	C		broad bean, fava bean	0.08	Se	Ethnoveterinary: Fodder given alone or with barley as a galactagogue for livestock	3	75%	—	—

Taxa & Voucher Code	St	Local Dialect Name	English Common Name	UV_c	PU	Local preparation and use	U_{is}	FL	PC	Different Uses Reported in PC
						Otolaryngological/Respiratory: Dried fava beans are boiled for a long time with carob fruits and then mixed with sugar and honey. Taken internally for cough.	1	25%	—	—
**Fagaceae**										
Quercus ilex L. subsp. *ilex*	W	arvulu di ghianda; anzine	evergreen oak; holly oak	0.04	Fr	Food: Acorns used to be dried and then toasted, then used as a coffee substitute [past use]	1	—	—	—
					Br	Household: Boiled to dye cotton fishing nets a darker color	1	—	—	—
**Iridaceae**										
Crocus longiflorus Raf.	W	cipudduzzi	yellow-throated crocus	0.15	Sti	Food: Used as a food colorant like saffron (found at high elevations)	5	71%	—	—
					Bu	Food: Eaten raw like an onion, with bread, and given to children. Most commonly used as food during times of famine.	2	29%	—	—
**Juncaceae**										
Juncus acutus L. subsp. *acutus*	W	giuncu; iuncu	spiny rush	0.33	AP	Maritime: Used to weave fish traps	6	38%	—	Urological: diuretic, Se [1, 2]
						Household: Used to weave ferret carriers for hunting	2	13%	—	—
						Household: Used to weave baskets for making ricotta and other local cheeses	8	50%	[1, 2]	—
**Lamiaceae**										
Clinopodium nepeta (L.) Kuntze subsp. *nepeta* CQ-627	W	nipitedda; menta sarbaggia;	lesser calamint	0.13	Le	Food: Seasoning for fish, especially tuna. The local dish "ammuntonato" is made with the leaves and garlic inserted	5	83%	—	Respiratory: respiratory disease, Le [1, 2, 4]

Taxa & Voucher Code	St	Local Dialect Name	English Common Name	UV_c	PU	Local preparation and use	U_{is}	FL	PC	Different Uses Reported in PC
						in cuts in the meat before cooking.				
						Gastrointestinal: Tea is drunk to treat stomachache as a digestive stimulant.	1	17%	[3, 4]	—
Salvia rosmarinus Schleid. [= *Rosmarinus officinalis* L.]	W; C	rosamarina	rosemary	0.38	Wo	Maritime: Boiled and used to dye white fishing nets	1	6%	—	—
						Household: Firewood	4	22%	—	—
					AP	Food: Used to season/flavor a variety of dishes	11	61%	[1-3]	—
					Le	Gastrointestinal: Decoction is drunk as a digestive aid and liver cleanse	1	6%	[1-4]	Respiratory: respiratory disease, cough, Le [2, 4]
						Gastrointestinal: Decoction prepared with a pinch of rosemary leaves, bay leaves, malva, 2 leaves of sage, 2 leaves of calamint and 2 prickly pear flowers. This is drunk as a digestive stimulant.	1	6%	—	—
Salvia officinalis L. subsp. *officinalis*	C	—	common sage	0.13	Le	Gastrointestinal: Decoction prepared with 2 leaves of sage, a pinch of rosemary leaves, bay leaves, malva, 2 leaves of calamint and 2 prickly pear flowers. This is drunk as a digestive stimulant for treating stomach pains.	2	33%	[2]	Dermatological: Skin disease, Le [2]
						Food: Seasoning for tuna dishes	4	67%	[1, 2]	—
Thymbra capitata (L.) Cav. CQ-610	W	sataredda; satareddu	conehead thyme	0.54	AP	Household: Honey plant	1	4%	—	Gastrointestinal: eupeptic,

Taxa & Voucher Code	St	Local Dialect Name	English Common Name	UV_c	PU	Local preparation and use	U_{is}	FL	PC	Different Uses Reported in PC
										stomachic, AP [3, 4]
						Food: To flavor fish when conserving them under salt (sardines, tuna, mackerel)	3	12%	—	—
						Food: Seasoning for various dishes. The flavor is imparted by adding the dry parts to the fire and the resulting smoke flavoring the food.	4	15%	[1, 2]	—
						Food: Seasoning for various dishes. Dried in the sun and chopped up small for use as a spice.	7	27%	—	—
						Household: To light fires and perfume the hearth (use dry aerial parts)	7	27%	—	—
						Food: Flavoring for grappa (steeped in the alcohol to impart aroma)	3	12%	—	—
						Otolaryngological/Respiratory: Decoction for cough (anti-tussive)	1	4%	[1-4]	
**Lauraceae**										
Laurus nobilis L.	C	addauru	laurel; sweet bay	0.69	Le	Gastrointestinal: Tisane drunk for treating stomachache and as a digestive aid; sometimes mixed with sugar.	11	33%	[1, 2]	Food: aromatic herb, Le [1, 2]
						Gastrointestinal: Digestive aid used especially for young children to help them burp (older children were given bicarbonate)	5	15%	—	—
						Gastrointestinal: Infusion with *Aloysia* to treat stomachache	2	6%	—	—

Taxa & Voucher Code	St	Local Dialect Name	English Common Name	UV_c	PU	Local preparation and use	U_{is}	FL	PC	Different Uses Reported in PC
						Gastrointestinal: Leaves are combined with lemon rind and made into a decoction for stomachaches	6	18%	—	—
						Otolaryngological/Respiratory: Syrup for cough (anti-tussive) made with bay leaves, lemon leaves, dried figs and carob pods.	4	12%	—	—
						Dermatological: Leaves mixed with hot bread and garlic, topically applied to skin infections (boils, cysts, furuncles)	4	12%	—	—
					WP	Household: Used as firewood in the home	1	3%	—	—
**Linaceae**										
Linum usitatissimum L. subsp. *angustifolium* (Huds.) Thell. [=*Linum bienne* Mill.]	W	linusa; linosa	pale flax	0.08	Se	Dermatological: Blanched in boiling water and made into a cataplasm for topical applications to contusions or furuncles	2	50%	—	Dermatological: emollient; Gastrointestinal: laxative, Se [3]
						Otolaryngological/Respiratory: Blanched in boiling water and made into a cataplasm for topical application to the chest in treating bronchitis	2	50%	—	—
**Lythraceae**										
Punica granatum L.	C	ranatu	pomengranate	0.21	Fr, Se	Ethnoveterinary: Fodder for hens. The fruits damaged by birds were used for this.	5	50%	—	—
						Food: Juice drunk.	4	40%	[1-3]	Urological: diuretic; Gastrointestinal:

Taxa & Voucher Code	St	Local Dialect Name	English Common Name	UV_c	PU	Local preparation and use	U_{is}	FL	PC	Different Uses Reported in PC
										astringent, AP [3, 4]
					St	Household: to weave (along with cane stems and young olive branches) ferret carriers for hunting.	1	10%	[1, 2]	—
**Malvaceae**										
Gossypium hirsutum L.	C	cuttuni	upland cotton	0.02	Fr	Household: [Cultivated for textiles and clothing]	1	—	—	—
Malva arborea (L.) Webb & Berthel. CQ-636	W	marva; panuzzi	tree mallow	0.54	Fr	Food: Snack food. Eaten as part of childhood games. Also known as "*Panuzzi i San Giuseppi*".	7	27%	—	—
					Fl	Dermatological: Fresh leaves are pounded with salt and topically applied to treat skin abscesses and furuncles.	1	4%	—	—
						Oral health: A decoction of dry flowers is topically applied to treat tooth abscesses	1	4%	—	Similar uses reported for *Malva sylvestris* [2]
					Le	Oral health: Boiled with a small amount of water, and then olive oil and bread crumbs are added and kneaded together. This is used as a cataplasm for treating abscess in the mouth (e.g. oral infections and tooth abscesses).	1	4%	—	—
						Oral health: Fresh young leaves are mashed and topically applied to the gingiva to treat tooth abscesses. Some report that the leaf mash is	2	8%	—	—

Taxa & Voucher Code	St	Local Dialect Name	English Common Name	UV_c	PU	Local preparation and use	U_{is}	FL	PC	Different Uses Reported in PC
						wrapped in gauze before applying.				
						Dermatological: Fresh leaves are pounded with salt and topically applied to treat skin abscesses and furuncles	1	4%	—	—
						Dermatological: Boiled with a small amount of water, and then olive oil and bread crumbs are added and kneaded together. This is used as a cataplasm for the topical treatment of skin abscesses and boils.	1	4%	—	—
						Ethnoveterinary: A decoction of leaves (2 liters) is mixed with olive oil (1 liter) and fed to cattle that suffer from a digestive block twice a day until resolved	1	4%	—	—
						Gastrointestinal: Decoction is drunk as a refreshing beverage to treat intestinal ailments	2	8%	—	—
					Fl, Le	Gastrointestinal: Decoction is drunk as a refreshing beverage to treat intestinal ailments	1	4%	—	—
						General health: Decoction drunk as anti-inflammatory	1	4%	—	—
						Oral health: A tisane of leaves and flowers is topically applied (as a mouth rinse) for gingival inflammation	3	12%	—	—

Taxa & Voucher Code	St	Local Dialect Name	English Common Name	UV_c	PU	Local preparation and use	U_{is}	FL	PC	Different Uses Reported in PC
					AP	Gastrointestinal: An infusion of aerial parts is drunk to treat stomachache	2	8%	—	—
						Oral health: Decoction topically applied to treat gingival inflammation	1	4%	—	—
						Optometric: Decoction topically applied to treat eye inflammations (conjunctivitis)	1	4%	—	—
Malva multiflora (Cav.) Soldano, Banfi & Galasso	W	marva; marva servatica	Cretan malva	0.35	Le	Oral health: Decoction used as a mouth rinse to treat toothache from tooth abscess	1	6%	—	—
						Gastrointestinal: Decoction drunk as a digestive aid to treat stomachache	1	6%	—	—
						Musculoskeletal/Neurological: Poultice topically applied to treat contusions and edema as an anti-inflammatory	1	6%	—	—
					Fl, Le	Gastrointestinal: Dried leaves and flowers used in decoction drunk as a digestive aid (laxative) for stomach pains	4	24%	—	—
						General health: Decoction drunk as anti-inflammatory	1	6%	—	—
					Fr	Food: Snack food. Eating as part of childhood games like a candy. Also known as "*Panuzzi i San Giuseppi*" or "*Pani di San Giuseppe*".	6	35%	—	—
					AP	Oral health: A pack soaked in the decoction of the aerial parts is topically applied to treat tooth abscess	1	6%	—	—
						Optometric: A pack soaked in the decoction of the aerial	1	6%	—	—

Taxa & Voucher Code	St	Local Dialect Name	English Common Name	UV_c	PU	Local preparation and use	U_{is}	FL	PC	Different Uses Reported in PC
						parts is topically applied to treat eye inflammations (conjunctivitis)				
						Oral health: A cataplasm of mashed aerial parts topically applied to gingival inflammations	1	6%	—	—
Malva nicaeensis All.	W	—	bull mallow; French mallow	0.02	Fl, Le	General health: Decoction drunk as anti-inflammatory	1	—	[1-3]	Veterinary applications [1, 2]
**Moraceae**										
Ficus carica L.	C	ficu	fig	0.19	Fr	Otolaryngological/Respiratory: Fruit (fresh or dry) is prepared into a syrup by mixing with lemon leaves, bay leaves, and carob fruits; taken internally as an anti-tussive	6	67%	[1-4]	Dermatological: treat warts, La [2-4]; Gastrointestinal: laxative, Fr [3, 4].
						Food: Fruits are speared with a wooden stick and sun dried or oven dried for storage and used as an ingredient in various desserts; sometimes also with carob fruits	2	22%	[1, 2]	—
						Food: In marmalades	1	11%	—	—
Morus alba L.	C	cevusu jancu	white mulberry	0.04	Fr	Food: Eaten raw as a snack	1	—	—	
						Food: Boiled down into a syrup, used in drinks	1	—	—	—
Morus nigra L.	C	cevusu nivuru	black mulberry	0.04	Fr	Food: Eaten raw as a snack	1	—	—	—
						Food: Boiled down into a syrup, used in drinks	1	—	—	—
**Myrtaceae**										
Myrtus communis L. CQ-639	W	murtidda; murteddu	common myrtle	0.15	Fr	Food: Eaten raw along with "zizzole" (*Ziziphus jujuba*)	2	29%	—	Gastrointestinal: astringent, stomachic, Le, St

Taxa & Voucher Code	St	Local Dialect Name	English Common Name	UV_c	PU	Local preparation and use	U_{is}	FL	PC	Different Uses Reported in PC
										[3, 4]; Dermatological: cosmetic, powder for skin, Le [1, 2]
					Br	Household: For the festival of San Giuseppe (March 19), it is customary to collect branches and decorate homes and boat prows with them	4	57%	—	—
						Maritime: Straight branches are used to make a "*cucedda*" to repair fishing nets	1	14%	—	—
**Oleaceae**										
Olea europaea L. var. *europaea*	C; W	aliva	olive tree	0.29	Le	Cardiovascular: 40 small leaves (about 20 g) are boiled in 1 liter of water for 5 minutes, then drunk in the morning and evening to lower blood pressure to manage hypertension	5	36%	[3, 4]	Urological: diuretic; General health: febrifuge, Le [3, 4]
						General Health: 40 small leaves (about 20 g) are boiled in 1 liter of water for 5 minutes, then drunk in the morning and evening to manage diabetes	4	29%	—	—
					Oil	Dermatological: Olive oil is topically applied as an emollient to treat insect stings (especially from wasps)	1	7%	[4]	—
						Household: A drop of olive oil was added to a dish of water with the fingertip. If the oil spreads on the plate, then this is an indication of the presence	1	7%	—	—

Taxa & Voucher Code	St	Local Dialect Name	English Common Name	UV_c	PU	Local preparation and use	U_{is}	FL	PC	Different Uses Reported in PC
						of Evil Eye. To cure this, prayers and then a sign of the cross must be made on the patient's head in a process called "*livari l'occhiu*". The oil is used as a diagnostic tool.				
					St	Household: Young stems/branches are used along with the stems of giant reed (*Arundo donax*) and pomegranate (*Punica granatum*) to to weave ferret carriers for hunting)	1	7%	[1, 2]	—
						Household: The young stems were woven to produce baskets and fish traps (*nasse*).	1	7%	[1, 2]	—
					Wo	Household: Firewood	1	7%	—	—
Phillyrea latifolia L. CQ-608	W	—	green olive tree; mock privet	0.02	Wo	Household: Firewood	1	—	—	Urological: diuretic, tonic; General health: tonic, Le [3]
**Orobanchaceae**										
Orobanche crenata Forssk.	W	lupa	bean broomrape	0.10	WP	Nuisance: If it grows near fava beans in the garden, it kills them	5	100%	—	—
**Oxalidaceae**										
Oxalis pes-caprae L.	W	cicireddu; acitu forti	Bermuda buttercup	0.06	St	Food: Sucked on as a snack to taste/enjoy the sour flavor	3	—	[1]	Urological: diuretic, depurative, Le [1, 3]; Nuisance: toxic to animals [1]
**Papaveraceae**										

Taxa & Voucher Code	St	Local Dialect Name	English Common Name	UV_c	PU	Local preparation and use	U_{is}	FL	PC	Different Uses Reported in PC
Glaucium flavum Crantz CQ-588; CQ-908	W	cavulu marinu	yellow horned poppy; sea-poppy	0.79	Le	Musculoskeletal/Neurological: Fresh leaves pounded with salt to make a cataplasm and topically applied to treat hematomas/contusions. Anti-inflammatory. (A few report also adding olive oil. Can also store in bottles after blending.)	26	68%	[1, 2]	—
						Dermatological: Fresh leaves are pounded (without salt) to make a cataplasm which is topically applied to burn wounds	3	8%	—	—
						Musculoskeletal/Neurological: Fresh leaves are pounded with salt to make a cataplasm and topically applied to treat swelling (oedema) from dislocations and sprains. Anti-inflammatory.	8	21%	—	—
					Fl	General health: Infusion of dried flowers has psychoactive effects	1	3%	—	—
Papaver rhoeas L. subsp. *rhoeas* CQ-567	W	papaveru; paparina	common poppy; corn poppy; field poppy	0.25	Fl, Fr	General health: Infusion drunk as a calmative agent for nervous disorders or difficulty sleeping (among children). Long-term exposure in childhood is thought to cause sterility in men in adulthood.	12	100%	[1-4]	Respiratory: bechic; General health: diaphoretic, Fl [4]
**Poaceae**										
Ampelodesmos mauritanicus (Poir.) T. Durand & Schinz CQ-645	W	disa	Mauritiana grass; rope grass	0.31	Le	Household: Leaf fibers are beaten and braided/woven in the production of baskets	3	20%	—	—

Taxa & Voucher Code	St	Local Dialect Name	English Common Name	UV_c	PU	Local preparation and use	U_{is}	FL	PC	Different Uses Reported in PC
						Maritime: Leaf fibers are beaten and braided/woven in the production of rope for use by fishermen	2	13%	—	—
						Maritime: Leaf fibers are beaten and braided/woven in the production of fishing nets	4	27%	—	—
						Household: Not common on the island of Levanzo, and was acquired by trade with the mainland (Trapani)	1	7%	—	—
						Household: Leaves were folded and attached to a cane (*Arundo donax*) with a nail to produce a tool called "*mazzu*" (known as "*zurbu*" in Trapani). This is used to mix the milk when making ricotta.	1	7%	—	—
						Household: Used to construct a roof or top for covering haystacks.	1	7%	—	—
					St	Household: Stems used to roll/form the shape of a traditional pasta shape "*busiate*"; used when eating a dish of wild fennel and tuna	3	20%	—	—
Arundo donax L. CQ-631	W	canna	giant reed	0.25	No	Dermatological: The white membrane found at the node of the stem is extracted and topically applied to bleeding lacerations as a hemostatic	4	33%	—	Urological: diuretic, depurative; General health: diaphoretic, Rh [3, 4]
					St	Household: Large stems (in diameter of the cane) are used to make "*u cannolu*" (a big	2	17%	—	—

Taxa & Voucher Code	St	Local Dialect Name	English Common Name	UV_c	PU	Local preparation and use	U_{is}	FL	PC	Different Uses Reported in PC
						straw) for pouring wine from barrels				
						Household: Stems are tied together to make a broad mat for providing shade, locally known as "*friscale*"	2	17%	[1, 2]	—
						Household: To weave (along with young olive and pomegranate branches) ferret carriers for hunting	1	8%	—	—
						Household: The large segments of the cane stem are used to make the form of "*cannoli*", a classic Sicilian dessert	1	8%	—	—
					WP	Household: Considered a plant that is blessed by God due to its great utility	2	17%	—	—
Avena spp.	W	ina		0.17	Fr	Household: The spiky fruits were pulled off of the plant and thrown onto clothes as a game. If the fruit sticks, this is a sign that the person would become engaged/married.	2	25%	—	—
						Household: The spiky fruits were pulled off of the plant and thrown onto clothes as a game. The number of fruits that stick indicates the number of girlfriends a boy would have.	2	25%	—	—
					St	Household: After removing the spiky fruit, a small loop remains at the apical end. This – using slow movements – can be used to capture lizards by	1	13%	—	—

Taxa & Voucher Code	St	Local Dialect Name	English Common Name	UV_c	PU	Local preparation and use	U_{is}	FL	PC	Different Uses Reported in PC
						looping it around the lizard head.				
					AP	Dermatological: Blanched in warm water and topically applied to treat boils	1	13%	—	—
						Dermatological: Blanched in warm water and topically applied to treat mosquito bites	1	13%	—	—
						Ethnoveterinary: Oats fed to livestock if suffering from stomach problems	1	13%	—	—
Avena sativa L. subsp. *sativa*	C	—	oat	0.21	Se	Ethnoveterinary: Seeds used as animal feed, to augment milk production as a galactagogue	5	50%	—	Respiratory; Musculoskeletal: anti-rheumatic; Urological: diuretic, Se [4]
						Ethnoveterinary: Bran soaked in water and fed to animals as a nutritional feed supplement	5	50%	—	—
Cynodon dactylon (L.) Pers.	W	ramigna; gramigna	Bermuda grass	0.35	Rh	Ethnoveterinary: Boiled with leaves of *Borago officinalis* and then a bit of olive oil is added. Given to livestock (cattle and sheep) suffering from digestive afflictions to drink.	2	12%	—	—
						Urological: A decoction of rhizomes (dried 2-3 days) is prepared and drunk as a refreshing diuretic beverage to treat infections and inflammations of the urinary tract	14	18%	[1-4]	—
					WP	Dermatological: An infusion is used as an external	1	6%	[3]	—

Taxa & Voucher Code	St	Local Dialect Name	English Common Name	UV_c	PU	Local preparation and use	U_{is}	FL	PC	Different Uses Reported in PC
						rinse/liniment to treat irritated skin; soothing to the skin				
Dasypyrum villosum (L.) Borbás	W	—	mosquito grass	0.08	Se	Ethnoveterinary: Fodder for chickens, often mixed with corn	1	25%	—	—
						Ethnoveterinary: Fodder for cattle, mixed with typical feed or with fava beans; used as a galactagogue	3	75%	—	—
Hordeum vulgare L. subsp. *vulgare*	C	—	common barley	0.02	Se	Food: [Toasted and ground as a coffee substitute]	1	—	—	—
Hyparrhenia hirta (L.) Stapf subsp. *hirta*	W	fanusu	thatching grass	0.04	AP	Ethnoveterinary: Fields are burned to foster growth of fresh shoots after rain (about 15 days) for sheep to forage on; also provided as fodder for livestock	2	—	—	—
Triticum turgidum L. subsp. *durum* (Desf.) Husn. – [=*Triticum durum* Desf.]	C	furmentu	durum wheat	0.15	Se	Ethnoveterinary: Cows are forced to eat it by tying a bowl of the grain to the snout in order to promote labor and eliminate the placenta following birth	2	29%	—	—
						Ethnoveterinary: Fodder for chickens, given along with corn and barley	1	14%	—	—
						Dermatological: An infusion of the bran is added to the child's bath as a refreshing and anti-reddening therapy for irritated skin. This is used for infants and young children.	4	57%	—	—
Zea mays L. subsp. *mays*	C	—	corn	0.02	Se	Ethnoveterinary: Fodder for hens, mixed with wheat and barley	1	—	—	—

Taxa & Voucher Code	St	Local Dialect Name	English Common Name	UV_c	PU	Local preparation and use	U_{is}	FL	PC	Different Uses Reported in PC
**Portulacaceae**										
Portulaca oleracea L. CQ-900	W	pucciddana; purciddana	common purslane	0.19	Le	Food: Tender leaves eaten raw or in salads	8	89%	[1, 2]	Urological: diuretic, depurative; Gastrointestinal: vermifuge; General Health: anti-scurvy, anti-inflammatory, AP [3, 4]
						General health: Eaten raw, considered good for the stomach and skin health	1	11%	—	—
**Potamogetonaceae**										
Posidonia oceanica (L.) Delile CQ-606	W	arga; triscia; ammariune	Mediterranean tapeweed; neptune grass	0.06	Le	Dermatological: Leaves are blanched in hot water and then inserted into the rectum at night to treat hemorrhoids	2	—	—	—
						Dermatological: Leaves are blanched in hot water and then mixed with bread crumbs and chopped tomato and topically applied as a suppurative to mature skin boils and abscesses	1	—	—	—
**Pteridaceae**										
Adiantum capillus-veneris L.	W	capiddu venniri	maidenhair fern; southern maidenhair fern	0.06	Fro	Urological: Fronds are boiled in a decoction and then drunk as a diuretic – especially for children suffering from renal problems	3	100%	—	Gastrointestinal: eupeptic; Respiratory: respiratory disease; General Health: diaphoretic, Fro [3, 4]; Dermatological:

Taxa & Voucher Code	St	Local Dialect Name	English Common Name	UV_c	PU	Local preparation and use	U_{is}	FL	PC	Different Uses Reported in PC
										cosmetic, Fro [4]; Women's Health: abortifacient, Fro [1, 2]
**Rhamnaceae**										
Rhamnus lycioides L. subsp. *oleoides* (L.) Jahand. & Maire CQ-605	W	spinaripuci; spinaporci	black hawthorn; European buckthorn	0.19	Wo	Household: Firewood, also used to heat bread baking ovens	9	100%	—	—
Ziziphus jujuba Mill.	C	zizzuli	jujube red date; Chinese date	0.08	Fr	Food: Eaten raw with *Myrtus communis* fruits	4	100%	—	—
**Rosaceae**										
Crataegus laevigata (Poir.) DC. CQ-864	W	azzalore	hawthorn	0.04	Fr	Food: Eaten raw, like a small sweet apple	2	-	—	—
Cydonia oblonga Mill.	C	cutugnu	common quince	0.10	Fr	Food: Jam or marmalades made in traditional terracotta mols	5	100%	—	—
Prunus dulcis (Mill.) D.A.Webb	C	mennula	almond	0.02	Se	Food: Ground into a flour and used to make sweets	1	-	—	—
**Rutaceae**										
Citrus×aurantium L.	C	aranciu amaru	bitter orange	0.08	Fr	Food: Made into traditional marmalades	4	100%	—	—
Citrus×limon (L.) Osbeck	C	limuni	lemon	0.33	Fr	Otolaryngological/Respiratory: Lemon is mixed with bay leaves and carob fruits to make a syrup for treating coughs (antitussive)	4	25%	—	—
						Gastrointestinal: Decoction of the lemon rind and *Aloysia citriodora* leaves drunk to treat gastritis	1	6%	—	—
						Gastrointestinal: Decoction of the lemon rind and bay leaves	1	6%	—	—

Taxa & Voucher Code	St	Local Dialect Name	English Common Name	UV_c	PU	Local preparation and use	U_{is}	FL	PC	Different Uses Reported in PC
						drunk as a digestive aid to treat stomachaches				
						Gastrointestinal: A decoction of 1 slice of lemon into a glass of water is used to treat digestive problems and stomachaches (sometimes sweetened with sugar)	5	31%	—	—
						Gastrointestinal: Half a lemon is juiced and mixed with a small spoon of olive oil. [Consumed to treat infections of the stomach, high fever, or diarrhea with green mucous during times of war].	1	6%	—	—
						Musculoskeletal/Neurological: Slice of fresh fruit put directly on the forehead to reduce fever	1	6%	—	—
						Musculoskeletal/Neurological: Coat slice of the lemon fruit with bicarbonate and topically apply to the forehead to treat headache or fever	2	13%	—	—
					Fr, Le	General Health: A decoction is prepared from 2 lemon leaves or the rind of half a lemon and drunk once a day for general health and wellbeing	1	6%	—	—
Ruta chalepensis L. CQ-585	W	ruta; aruta	fringed rue	1.08	Fr	Oral health: Topically applied to dental caries (for toothache). The fruit is used in a similar way to cloves for this purpose.	1	2%	—	—
					AP	Gastrointestinal: Infusion in grappa – the aerial parts are steeped in the alcohol to	3	6%	—	—

Taxa & Voucher Code	St	Local Dialect Name	English Common Name	UV_c	PU	Local preparation and use	U_{is}	FL	PC	Different Uses Reported in PC
						impart flavor. Drunk as a digestive aide.				
						General health: The smell is inhaled as an aromatherapy for nervous anxiety disorders or a sudden shock or scare	6	12%	—	General health: internal use of decoction for anxiety, AP [1]
						General health: To overcome symptoms of drunkenness or hangover, the plant is kept in pockets and smelled (aromatherapy)	6	12%	—	—
						General health: To manage anxiety, the plant is kept in women's pockets, or in a small sack held in the bodice or bras. The smell (aromatherapy) is relaxing.	7	13%	—	—
						Gastrointestinal: Topical poultice onto the abdomen as a vermifuge. Sometimes prepared with the plant crushed with a bread loaf.	4	8%	[1]	—
						Gastrointestinal: Topical poultice onto the abdomen to treat digestive troubles and stomach pains.	3	6%	—	—
						Gastrointestinal: Chew on the fresh aerial parts in small quantities to treat digestive problems.	2	4%	—	—
						Ethnoveterinary: Fresh plant (aerial parts) wrapped around wounds on donkeys for wound healing	2	4%	—	—
						Musculoskeletal/Neurological: Poultice topically applied to	1	2%	—	—

Taxa & Voucher Code	St	Local Dialect Name	English Common Name	UV_c	PU	Local preparation and use	U_{is}	FL	PC	Different Uses Reported in PC
						treat osteoarthritic and rheumatic pains.				
						Food: Flavoring for grappa (steeped in the alcohol to impart flavor)	5	10%	—	—
					Le	Gastrointestinal: Infusion as a vermifuge	1	2%	[1-4]	—
						Gastrointestinal: Topical poultice of boiled leaves onto the abdomen as a vermifuge	4	8%	—	—
						Gastrointestinal: Topical poultice of boiled leaves onto the abdomen to treat digestive troubles and stomach pains.	5	10%	—	—
					WP	General health: Panacea; local saying: "*la ruta tutti i mali astuta*" (the rue cancels any type of disease)	2	4%	—	Women's health: emmenagogue; General health: tonic, rubefacient, Le [3, 4]
**Scrophulariaceae**										
Verbascum sinuatum L. CQ-590	W		wavyleaf mullein	0.13	St, Br	Household: Stems and dried branches used to create brooms	6	100%	[1, 2]	Gastrointestinal: antispastic, bechic; General health: diaphoretic; Respiratory: expectorant; Urological: diuretic, Le, Fl [3]
**Solanaceae**										
Mandragora autumnalis Bertol. CQ-906	W	mannaraota	mandrake	0.10	WP	Nuisance: Poisonous. Leaves sometimes confused for those	5	100%	—	Musculoskeletal: articular pain, Fr [1, 2];

Taxa & Voucher Code	St	Local Dialect Name	English Common Name	UV_c	PU	Local preparation and use	U_{is}	FL	PC	Different Uses Reported in PC
						of the edible borage leaves (*Borago officinalis*)				Gastrointestinal: antispasmodic, Ro [3]
Nicotiana glauca Graham CQ-612	W	sucameli	tree tobacco	0.10	Le	Dermatological: Cataplasm of leaves topically applied to treat hemorrhoids	2	40%	—	—
						Dermatological: Leaves are blanched in water and then put on a cloth to cool. A small amount of olive oil is added, and then this is topically applied throughout the night to treat hemorrhoids.	1	20%	—	—
					Fl	Food: The floral nectar is sucked as a sweet snack, especially during childhood	2	40%	[1]	—
Solanum nigrum L. CQ-919	W	sulatra	American black nightshade	0.10	Fr	Dermatological: Poultice of fresh fruit applied to large infected boils (especially for large ones found on the neck); acts as a suppurative to mature the boil	4	80%	—	—
					Le	Dermatological: Poultice applied to large infected boils (especially for large ones found on the neck); acts as a suppurative to mature the boil.	1	20%	—	—
Solanum linnaeanum Hepper & P.-M.L.Jaeger CQ-623	W		devil's apple	0.02	Ro	Household: Rootstock were used for growing aubergine (*Solanum melongena*), but the resulting fruits were small and bittersweet [past use]	1	—	—	—
Solanum tuberosum L.	C	—	potato	0.04	Tu	General health: A slice of potato tuber is placed on the forehead as a febrifuge	1	—	—	—

Taxa & Voucher Code	St	Local Dialect Name	English Common Name	UV_c	PU	Local preparation and use	U_{is}	FL	PC	Different Uses Reported in PC
						General health: A slice of potato tuber is placed on the forehead to treat headache	1	—	—	—
Solanum villosum Mill.	W	sulatra	hairy nightshade; red nightshade	0.04	Fr	Dermatological: Fresh fruits crushed and topically applied to furuncles	1	—	—	—
					Le	Dermatological: Tender leaves crushed into a poultice and topically applied to furuncles	1	—	—	—
**Thymelaeaceae**										
Thymelaea hirsuta (L.) Endl. CQ-624	W	mufulena	shaggy sparrow-wort	0.19	AP, Wo	Household: Construction of brooms used to clean bread baking ovens	5	56%	—	—
						Household: Firewood and/or fire starter; less commonly used than *Pistacia lentiscus* for the same purpose due to lower abundance in the environment	4	44%	—	—
**Typhaceae**										
Typha angustifolia L.	W	pinna russeddu	narrowleaf cattail	0.17	Inf	Dermatological: Topically applied like a gauze bandage for bleeding wounds and lacerations, as a hemostatic and for wound healing. This is not available on the islands, but acquired on mainland in Trapani and stored dry in jars at home for ready use.	8	100%	[1, 2]	—
**Urticaceae**										
Parietaria judaica L. CQ-870	W	erba ventu	pellitory of the wall; spreading pellitory	0.15	AP	Household: Used to clean bottles	4	57%	—	—
						Dermatological: Pestled with salt and applied to contusions	1	14%	—	Dermatological: skin disease, Le [2]

Taxa & Voucher Code	St	Local Dialect Name	English Common Name	UV_c	PU	Local preparation and use	U_{is}	FL	PC	Different Uses Reported in PC
						Otolaryngological/Respiratory: Decoction drunk to treat whooping cough	1	14%		Similar use of leaves and roots reported for *P. officinalis* [4]
						Urological: Decoction drunk to treat renal calcoli	1	14%	[1, 4]	—
Urtica membranacea Poir. ex Savigny	W	ardicula; ardicola; firdicula	Mediterranean nettle	0.48	AP, Sh	Food: Young shoots or aerial parts eaten raw or boiled either alone, in salads or with rice	4	17%	—	—
					AP	Gastrointestinal: Aerial parts boiled and mixed with flour to make pasta, then eaten to treat stomachaches or as a food	2	9%	—	—
						Musculoskeletal/Neurological: Aerial parts are boiled, and the resulting herb water is used to soak the feet to treat inflammation and pain	1	4%	[3]	—
						Musculoskeletal/Neurological: Aerial parts boiled and then prepared as a cataplasm for application to painful or inflamed regions of the body	1	4%	—	—
						Musculoskeletal/Neurological: Aerial parts sieved, filtered and topically applied to reddened or inflamed areas of the body	1	4%	—	—
						Urological: Aerial parts prepared as a decoction, which is drunk as a diuretic. Recommended to only drink a small amount as it can be heating/irritating to the body.	8	35%	[3]	—

Taxa & Voucher Code	St	Local Dialect Name	English Common Name	UV_c	PU	Local preparation and use	U_{is}	FL	PC	Different Uses Reported in PC
						Ethnoveterinary: Aerial parts are fed to chickens to promote egg production	4	17%	—	—
						Household: Aerial parts are macerated in water, then sprayed on crops to protect them from parasites	1	4%	—	—
					WP	Dermatological: The fresh plant is rubbed against the skin until the skin is raw (almost to the point of bleeding) to treat skin infections of the hand (*rosuli*)	1	4%	—	Dermatological: dandruff treatment, WP [1]; decoction to protect against hair loss, Ro [2]
**Valerianaceae**										
Fedia graciliflora Fisch. & C.A.Mey.	W	peri–ri-ciocca	African valerian	0.38	Le	Food: Tender young leaves eaten raw in salads	16	89%	[1, 2]	—
						Cardiovascular: Leaves eaten to promote heart health	1	6%	—	—
						General health: Leaves eaten for calming and relaxant properties	1	6%	—	—
**Verbenaceae**										
Aloysia citrodora Palau CQ-910	C	citronella	lemon beebush	0.13	Le	Gastrointestinal: Infusion or decoction of leaves is combined with bay leaves (*Laurus nobilis*) and drunk to treat stomachaches and gastric upsets	2	33%	—	—
						Gastrointestinal: Decoction of leaves is combined with bay leaves, lemon rind and sage	3	50%	—	—
						Household: Insect repellent	1	17%	—	—
**Vitaceae**										
Vitis vinifera L.	C	racina	grapevine	0.06	Fr	Otolaryngological/Respiratory: Fruits are smashed and boiled	1	—	—	—

Taxa & Voucher Code	St	Local Dialect Name	English Common Name	UV_c	PU	Local preparation and use	U_{is}	FL	PC	Different Uses Reported in PC
						to make a caramelized syrup that is stored in the refrigerator and used throughout the winter as an antitussive				
						Food: Wine (*Vitis vinifera*) used to be diluted with water to make "peri", which was then mixed with a decoction of 4-5 carob fruits boiled in 4-5 liters of water. This is left to ferment and the volume reduces, yielding a final product of "carrubbata"	2	—	—	—

References

1. Bertolino F: **Indagine sugli usi tradizionali delle piante dell'Isola di Favignana (Egadi)**. Università degli Studi di Palermo; 1988.
2. Lentini F, Aleo M, Amenta R: **L'uso popolare delle piante nelle isole Egadi (Sicilia)** *Acta Phytotherapeutica* 1997, **4**(2):88-94.
3. Bonomo R, Trapani S: **Piante officinali nelle Egadi. Lavori dell'Istituto Botanico e del Giardino Coloniale di Palermo**. In., vol. 25. Biblioteca delle Scienze Agrarie, Palermo, Italy; 1974: 195-233.
4. Rizza U: **I Semplici di Favignana. Egadi e Natura, Litotipografia Abate, Paceco**. Italy; 2001.
